# Effect of Synthetic Vitamin A and Probiotics Supplementation for Prevention of Morbidity and Mortality during the Neonatal Period. A Systematic Review and Meta-Analysis of Studies from Low- and Middle-Income Countries

**DOI:** 10.3390/nu12030791

**Published:** 2020-03-17

**Authors:** Aamer Imdad, Faseeha Rehman, Evan Davis, Suzanna Attia, Deepika Ranjit, Gamael Saint Surin, Sarah Lawler, Abigail Smith, Zulfiqar A. Bhutta

**Affiliations:** 1 Department of Pediatric, Division of Pediatric Gastroenterology, Hepatology and Nutrition, SUNY Upstate Medical University, Syracuse, NY 13210, USA; aamer08@gmail.com (A.I.); faseha.21@gmail.com (F.R.); 2 Department of Public Health and Preventive Medicine, SUNY Upstate Medical University, Syracuse, Syracuse, NY 13210, USA; davisev@upstate.edu (E.D.); ranjitd@upstate.edu (D.R.); saintsug@upstate.edu (G.S.S.); 3 College of Medicine, University of Kentucky, Lexington, KY 40536, USA; suzango@gmail.com; 4 Health Science Library, SUNY Upstate Medical University, Syracuse, NY 13210, USA; lawlersa@upstate.edu (S.L.); smithab@upstate.edu (A.S.); 5 Centre for Global Child Health, The Hospital for Sick Children, Toronto, ON M5G 0A4, Canada

**Keywords:** Vitamin A, Dextrose, Probiotics, Synbiotics, Low- And Middle-Income Countries, Systematic Review, Neonate, Nutrition

## Abstract

*Background:* Suboptimal nutritional status of a newborn is a risk factor for short- and long-term morbidity and mortality. The objectives of this review were to assess the efficacy and effectiveness of neonatal synthetic vitamin A supplementation, dextrose gel and probiotic supplementation for prevention of morbidity and mortality during infancy in low and middle-income countries. *Methods:* We included randomized trials. Primary outcome was all-cause mortality. We conducted electronic searches on multiple databases. Data were meta-analyzed to obtain relative risk (RR) and 95% confidence interval (CI). Studies for vitamin A and Probiotics were analyzed separately. No studies were found for dextrose gel supplementation during neonatal period. The overall rating of evidence was determined by Grading of Recommendations Assessment, Development, and Evaluation (GRADE) approach. *Results:* Sixteen studies assessed the effect of vitamin A supplementation during the neonatal period. Based on pooled data from community-based studies only, there was no significant effect of vitamin A on all-cause mortality at age 1 month (RR 0.99, 95% CI 0.90, 1.08), 6 months (RR 0.98; 95% CI 0.89–1.08) and 12 months (RR 1.04, 95% CI 0.94, 1.14) but increased risk of bulging fontanelle (RR 1.53, 95% CI 1.12, 2.09). The overall quality of evidence was high for the above outcomes. Thirty-three studies assessed the effect of probiotic supplementation during the neonatal period and were mostly conducted in the hospital setting. Probiotics reduced the risk of all-cause mortality (RR 0.80, 95% CI 0.66, 0.96), necrotizing enterocolitis (RR 0.46, 95% CI 0.35, 0.59) and neonatal sepsis (RR 0.78, 95% CI 0.70, 0.86). The grade ratings for the above three outcomes were high. *Conclusions:* Vitamin A supplementation during the neonatal period does not reduce all-cause neonatal or infant mortality in low and middle-income countries in the community setting. Probiotic supplementation during the neonatal period seems to reduce all-cause mortality, NEC, and sepsis in babies born low birth weight and/or preterm in the hospital setting.

## 1. Introduction

The last two decades have seen a significant decline in rates of childhood mortality however the decline in the rate of neonatal (age 0–28 days) mortality has been slower than the decline in mortality beyond the neonatal period [1,2]. Globally, the percentage of neonatal mortality is the highest in South Asia and Sub Saharan Africa [1]. Optimal nutritional support during the neonatal period is vital to the short- and long-term survival of the newborn [3,4]. Poor nutritional status of neonates is a major cause of illness and can lead to poor growth, increased risk of infection, bleeding and neonatal death [3,4,5]. The risk of morbidity and mortality during the neonatal period is higher in low- and middle-income countries where many births happen at home and the prevalence of maternal malnutrition and incidence of low birth weight (birth weight less than 2500 g) and preterm birth (gestational age < 37 weeks) is high [3,6,7]. The most important nutritional intervention at birth is breastfeeding [4]. The topic of breastfeeding is covered in another manuscript of this supplement [8]. In this review, we focused on three nutritional interventions i.e. Vitamin A, Dextrose and Probiotics supplementation during the neonatal periods. 

World Health Organization estimates that there are 190 million children under five years of age who are vitamin A deficient. Synthetic vitamin A supplementation in children 6–59 months of age has been shown to reduce all-cause mortality and diarrhea specific mortality in low- and middle-income countries [9]. A similar effect, however, could not be demonstrated in trials where vitamin A was supplemented during the neonatal period especially in trials conducted in African countries [10]. We aimed to update the previous review on this topic [10] and include newer studies from the community and hospital setting from low and middle-income countries. 

Persistent neonatal hypoglycemia can cause significant morbidity and mortality during the neonatal period and beyond. Simple interventions like dextrose gel supplementation can help prevent and treat neonatal hypoglycemia in high risk newborn as shown in data from the developed countries [11,12]. The risk of persistent neonatal hypoglycemia might be higher in the low- and middle-income countries where a significant proportion of birth happens at home and a significant proportion of newborn babies are low birth weight and/or preterm [7]. We were interested to see if any of the studies conducted in low- and middle-income countries assessed the efficacy of dextrose gel for the prevention and treatment of neonatal hypoglycemia.

A Cochrane review published in 2014 showed that neonatal probiotic supplementation in low birth weight and preterm babies could decrease the risk of necrotizing enterocolitis [13]. Later studies and systemic reviews showed that probiotics can also decrease the risk of neonatal sepsis [14,15]. We aimed to update the previous reviews and conducted subgroup analyses that were not conducted in previous reviews. We also used the Grading of Recommendations Assessment, Development, and Evaluation (GRADE) methods to assess the overall quality of evidence, and this method was not used in the past reviews.

This paper is part of a series of reviews for Campbell collaboration and this manuscript presents the summary of a large systematic Campbell review conducted on this topic. Overall, the objectives of this review were to assess the efficacy and effectiveness of synthetic vitamin A supplementation, dextrose gel supplementation and probiotic supplementation for prevention of morbidity and mortality during infancy in low- and middle-income countries. We did not find any studies for dextrose gel supplementation so the focus of the review is mainly on vitamin A and probiotic supplementation. 

## 2. Materials and Methods 

The detailed methods were published in a protocol [16] and are available in the supplementary material document 1. Briefly, studies selected for inclusion in this review were either experimental or quasi-experimental studies that were designed as randomized control trials (RCTs). Other study designs were considered, such as before-after studies, regression discontinuity designs, interrupted time series (ITS) but none of these studies were included. We included neonates regardless of health status, including low birth weight and preterm infants; however, we excluded studies that focused on neonates with congenital anomalies. Studies were included that have multiple intervention arms, but only the arms that were eligible for this review were included in this study. We included studies only from low- and middle-income countries defined based world Bank criteria [17].

There were three different interventions that were studied in this review and they are as follows:

1. Neonatal vitamin A supplementation compared to no supplementation or placebo

2. Oral Dextrose gel supplementation during the neonatal period compared to no supplementation.

3. Neonatal oral probiotics/synbiotics supplementation compared to no probiotic supplementation/placebo.

For the vitamin A studies, only synthetic vitamin A supplementation was considered. The dose and frequency were not restricted and the comparison group was either a placebo or a normal standard of care.

For the oral dextrose studies, only dextrose gel was considered as the intervention and all other forms of dextrose administration were excluded. There were no restrictions on dose and frequency and the control group was either a placebo or a normal standard of care.

For the oral probiotics/snybiotics studies, there were no restrictions on dose or frequency. Studies that were done using prebiotics only were excluded. The control group was either a placebo or a normal standard of care.

Each of the aforementioned interventions were analyzed discretely and were not compared either directly or indirectly.

The primary outcomes measured in this review are as follows:

-All-cause neonatal mortality (death between 0–28 days of life)

-All-cause infant mortality at 6 months (death between 0 days to 6 months of life)

-All-cause infant mortality at 12 months (death between 0 days to 12 months life). 

In the event that the outcomes were not reported in the follow-up periods mentioned (e.g., 28 days, 6 months, and 12 months), we first contacted the authors to obtain this data. If that data were not available from the authors, the following actions were taken: Mortality within the first six weeks of life was included as neonatal mortality at day 28, between 3–6 months were included as 6 months, and between 9–12 months were included as 12 months. If there was not a clear follow-up, the mortality data from the longest follow-up was included.

The secondary outcomes measured in this review are as follows: 

-Sepsis specific mortality measured between 0–28 days, 0 days to 6 months and 0 days to 12 months of life

-Neonatal sepsis (as defined by authors) in the first six weeks of life

-Necrotizing enterocolitis (as defined by the authors)

-Vitamin A Deficiency

-Prevention of Hypoglycemia (as defined by authors) during the neonatal period

-Treatment of Hypoglycemia (recurrence of hypoglycemia after the episode treated)

-Any adverse reactions during the intervention period

-Serious adverse events

-Neurodevelopmental outcomes at 12 and 24 months and the longest follow-up

A neurodevelopment outcome is an event that involves any cognitive, neurologic, and/or sensory outcomes. 

Studies were identified using electronic and other sources and was not dependent on the outcomes of the interventions. The following databases were used for the electronic searches: PubMed, EMBASE, the Cochrane Library, Cochrane Central Register for Controlled trials, Web of Science, CINHAL, Scopus, LILACS, Popline, and WHO Global Health Library. The search strategies were available in the Appendix A. The searched were conducted on the following dates: Vitamin A: 12/10/18 (Updated on 11/13/19); Probiotics: 2-8-19 (updated on November 27, 2019); Dextrose: 4-25-19 (updated on Nov 26, 2019). There were no restrictions applied to the searches based on outcomes, study design, or language. There were also no restrictions on the date of publication. We did restrict the studies to Human Studies only. Searches for ongoing trials was conducted at ’www.clinicaltrials.gov’ and WHO’s ICTRP trials database. International agency websites were also searched, such as WHO (including WHO’s Reproductive Health Library), UNICEF, Global Alliance for Improved Nutrition, International Food Policy Research Institute, International Initiative for Impact Evaluation (3ie), Nutrition International, UNICEF, World Bank, USAID and affiliates (e.g. FANTA, SPRING) and the World Food Programme. Grey literature was searched by using the following resources: Nutrition International (NI), Global Alliance for Improved Nutrition (GAIN), International Food Policy and Research Institute (IFPRI), and WHO library database (WHOLIS). The reference sections of previously published reviews and the latest published studies were searched for potential studies of interest. 

Two authors independently screened the title/abstract of potential studies to include by using prespecified inclusion/exclusion criteria. Following the initial screening, the full text of the studies was reviewed using the same inclusion/exclusion criteria as the initial screening. If there was any conflict between the two authors over the inclusion of a study, a third reviewer was consulted to evaluate the study for inclusion. A web-based software system called ‘Covidence’ was used to screen titles, abstracts, and full text of the studies.

Data from the included studies were abstracted into standardized data abstraction forms for each intervention by two authors, and any discrepancies were resolved by discussion. The data extraction sheet had the following information for each of the interventions: General Study Information: authors, publication year, study design, study setting: city/town, urban/urban slum/rural/mixed setting, duration of data collection, date of data collection; study population: sample size recruited, sample size analyzed, female (%), description of participants (i.e. inclusion/exclusion criteria applied to recruitment); intervention characteristics: type of intervention, duration of intervention, unit of randomization (where applicable), dose, frequency of provision, duration of follow up, attrition rate; quality assessment; outcome, outcome Measure Treatment Group, outcome Measure Comparison Group, standard deviation, effect measure, 95% Confidence Interval.

Two authors independently assessed the risk of bias associated with each included study using the Cochrane’s Risk of Bias tool [18]. The risk of bias assessment included the following items: random sequence generation, allocation concealment, blinding of participants and personnel, blinding of outcome assessment, incomplete outcome data, selective reporting, and any other sources of bias.

We used the software review Manager 5.3 to conduct the statistical analysis [19]. For randomized trials, we followed the intention to treat analysis (ITT). If ITT was not available, and the author reported the analyses as specified in the protocol, we reconstructed the data to create an ITT analysis. We used a random effect model to account for expected heterogeneity in the intervention, comparisons, or setting within studies included in a given synthesis. We used the generic inverse variance method of meta-analysis for fixed effect models and random effect models. For Dichotomous outcome, relative risk was reported with 95% confidence interval (CI) and for continuous outcomes, mean difference was reported with 95% CI. For subgroup analysis, we used an interaction test to determine if there was a relevant difference in effect across subgroups. If the number of included studies for the intervention was more then 10, we used a funnel plot and its symmetry to evaluate for publication bias. If the funnel plot was indicative of publication bias, Egger’s test was used to further evaluate for publication bias [18]. Heterogeneity was assessed statistically by using Tau2, I2, significance of the Chi-square test, and by inspecting the forest plots visually. We assessed the quality of overall evidence using the GRADE approach. This method of quality assessment considers study type, within-study risk of bias (methodological quality), directness of evidence, heterogeneity, precision of effect estimates and risk of publication bias [20]. We rated the quality of the body of evidence for each key outcome as ’high’, ’moderate’, ’low’ or ’very low’ [20].

### 2.1. Subgroup Analysis & Investigation of Heterogeneity 

#### 2.1.1. Neonatal Probiotic Supplementation

1. Gestational Age: Full-Term and Preterm

2. Strains used in Probiotics: Single Strain vs. Multiple Strain and of type of strain used in each probiotic.

3. Strains used in Probiotics: Contains Lactobacillus vs. Bifidobacterium vs. Both

4. Settings: Community-Based vs. Hospital Setting

5. Type of Feedings: Breastmilk vs. Formula Milk vs. Mixed

#### 2.1.2. Neonatal Vitamin A Supplementation

We intended to perform numerous subgroup analyses for neonatal vitamin A supplementation; however, a recent IPD analysis [21] covered those analyses, so we decided not to perform any subgroup analysis for vitamin A supplementation.

#### 2.1.3. Oral Dextrose Gel Supplementation

There were no studies were found using dextrose gel supplementation, so no subgroup analysis was performed for this intervention.

### 2.2. Sensitivity Analysis 

Sensitivity analyses were performed as follows: 

-High quality studies vs Low quality studies. The quality of study will be based on risk of bias assessment

-Random vs Fixed effect models

Treatment of Qualitative Research

## 3. Results

### 3.1. Literature Search

**Figure 1:**
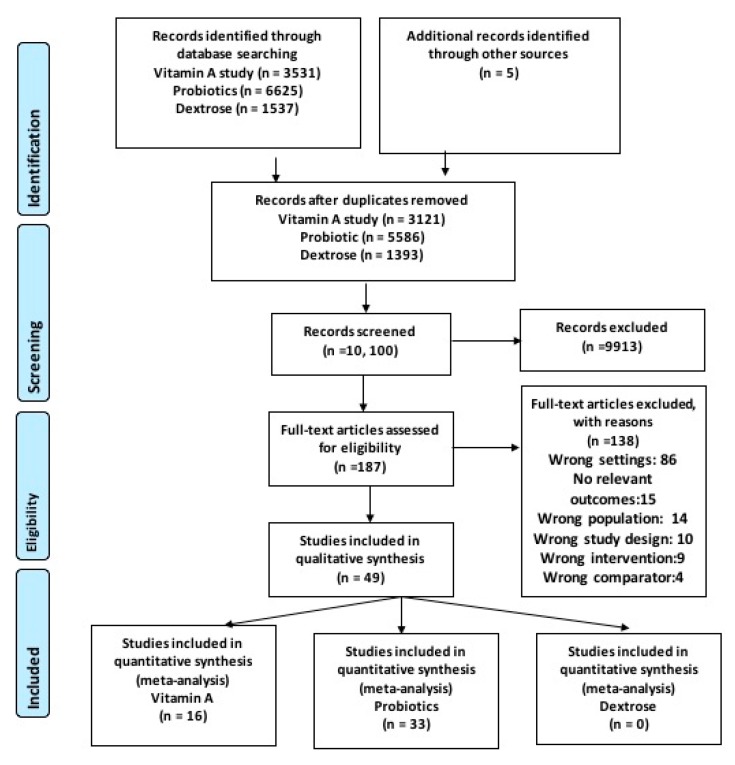
PRISMA flow Diagram.

Our literature yielded 3531 title for vitamin A, 6625 for probiotics and 1537 for dextrose supplementation. Figure 1 shows the results of literature search. We included sixteen studies for vitamin A supplementation [22,23,24,25,26,27,28,29,30,31,32,33,34,35,36,37], thirty three studies in probiotics supplementation and no study was found for dextrose gel supplementation. 

### 3.2. Characteristics of Included Studies

#### 3.2.1. Vitamin A Supplementation during the Neonatal Period 

A total of sixteen studies were included for vitamin A supplementation. Supplementary Table S1 gives details of included studies for neonatal vitamin A supplementation. Overall, all the included studies were randomized controlled trials. Thirteen studies were individually randomized [23,24,25,26,27,28,29,31,32,33,34,35,37] and 3 studies were cluster-randomized [22,30,36]. Three trials had multiple arms of interventions [24,25,31]. The included studies were conducted in 10 different countries with 4 studies in India [29,33,34,37], 3 studies conducted in Guiana Bissau [23,24,25], 2 studies in Bangladesh [28,30] and one each in Indonesia [27], Nepal [36], Ghana [26], Tanzania [32], Zimbabwe [31], China [35] and Pakistan [22]. Thirteen studies were conducted in the community settings while three studies were conducted in the hospital settings and included very low birth weight babies [29,35,37]. The median dose used in the included studies was 50, 000 IU. The control group in all the studies received placebo. 

#### 3.2.2. Probiotic Supplementation during the Neonatal Period 

A total of thirty three studies were included [18,38,39,40,41,42,43,44,45,46,47,48,49,50,51,52,53,54,55,56,57,58,59,60,61,62,63,64,65,66,67,68,69]. Supplementary Table S2 showed the characteristic of included studies for probiotic supplementation during the neonatal period. 

In summary, All the studies that evaluated the effect of probiotic supplementation during the neonatal period were individual randomized controlled trials. Two studies had multiple intervention groups [45,46]. One study included neonates with and without exposure to HIV (based on the maternal history of HIV) [55]. We included the data for these groups separately in the meta-analysis. Studies were conducted in 13 different countries with 9 studies conducted in India [18,46,49,54,57,59,64,66,68], 6 studies in Turkey [44,45,48,56,61,62], 3 studies each in Iran [38,43,63] and China [42,51,67], 2 each in South Africa [41,55], and Mexico [47,50] and one each in Brazil [39], Bangladesh [40], Colombia [58], Indonesia [53], Nepal [68], Pakistan [52] and Thailand [60]. All the studies were conducted in the hospital setting except three studies where participants were followed in the community settings [41,57,68]. Only one study [41] included neonates that were full term, and the rest of the studies included participants that were either low birth weight or preterm or both. Five studies used a preparation that had a probiotic + prebiotic (synbiotic) [41,45,48,54,57]. Sixteen studies used a placebo [42,43,44,45,46,53,56,57,58,59,64,68] and rest of the studies used a group with no probiotic supplementation but received standard of care.

#### 3.2.3. Risk of Bias 

Supplementary Figures S1 and S2 shows risk of bias in the included studies for vitamin A and probiotic supplementation during the neonatal period respectively. 

#### 3.2.4. Excluded Studies:

Supplementary Table S4 showed the table of excluded with reasons for exclusion. 

### 3.3. Effect of Interventions 

#### 3.3.1. Vitamin A Supplementation during the Neonatal Period 

##### All-cause Mortality

**Figure 2:**
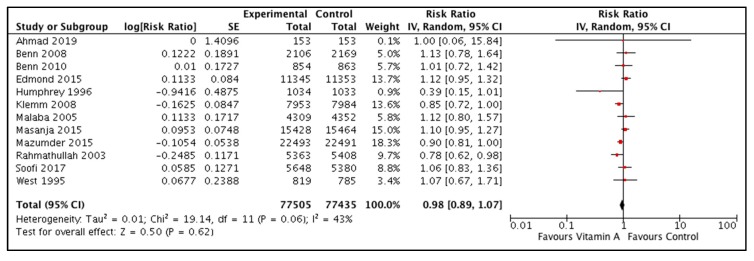
Effect of neonatal vitamin A supplementation on all-cause mortality at 6 months from studies conducted in the community settings from low- and middle-income countries.

We pooled the data for all-cause mortality from community-based studies only. Data were available from five studies for all-cause mortality at 1 month [22,26,30,32,33]. The pooled results did not show any significant difference for vitamin A supplemented group compared to control (RR 0.99, 95% CI 0.90, 1.08). Use of fixed effect model did not change the summary estimate for neonatal mortality (RR 0.99, 95% CI 0.90, 1.08). Twelve studies from community settings reported the data for effect of neonatal vitamin A supplementation on all-cause mortality at 6 months [22,23,24,26,27,28,30,31,32,33,34,36]. The combined results showed no significant difference between the intervention and control group [RR 0.98; 95 CI 0.89, 1.07) (Figure 2). Similarly, no difference in all-cause mortality was noted at 12 months (RR 0.98; 95 CI 0.89, 1.07) from pooled data from 8 studies [23,24,26,27,29,31,32,33]. 

Three studies were conducted in the hospital settings for newborns that were admitted to the neonatal intensive care unit [29,35,37]. Two studies [35,37] did not show an effect on all-cause mortality however there was a beneficial effect in one study [29] (RR 0.44, 95% CI 0.22–0.84).

Adverse outcome of Bulging Fontanelle was reported in six studies [23,26,27,28,32,33]. The pooled results showed a 53% increased risk in the intervention group compared to control (RR 1.53, 95% CI 1.12, 2.09). Data from five studies [23,26,27,32,33] showed no difference in incidence of vomiting for intervention vs. control group (RR 1.00, 95% CI 0.93, 1.07). 

Table 1 gives the GRADE rating of the primary outcomes and selected secondary outcomes. 

##### Neurodevelopment Outcomes

Two studies reported long-term neurodevelopment outcomes after use of vitamin A supplementation during the neonatal period [27,30]. As the outcomes measured and duration of follow up, were different, we did not pool the studies and describe the results from individual studies as follows

The study by Humphrey et al. [27] reported the data on neurodevelopment outcomes at 3 year follow up post-neonatal vitamin A supplementation. The study authors focused on data for children with and without bulging fontanelle who received vitamin A or placebo. The results showed that neonatal vitamin A supplementation did not have any adverse effect on development in the presence or absence of bulging fontanelle. Neonatal vitamin A supplementation had a positive effect on all developmental scores.

The study by Klemm et al. [30] reported data on neurodevelopment at 8-year post-neonatal vitamin A supplementation. The authors followed a cohort of participants who received neonatal vitamin A, or their mother was given vitamin A during pregnancy. The results showed no significant difference in intelligence, memory, and motor function; however, when the neonates and their mothers were supplemented with vitamin A versus placebo, it increased their performance in reading, spelling, and math computation.

##### Retinopathy of Prematurity 

One study reported that the incidence of retinopathy of prematurity [35]. The vitamin A supplemented group (1.6%) had lower rates of Type 1 retinopathy of prematurity compared to control (6.9%) and these results were statistically significant (p value 0.030)

#### 3.3.2. Probiotic Supplementation during the Neonatal Period 

##### All-cause mortality

**Figure 3:**
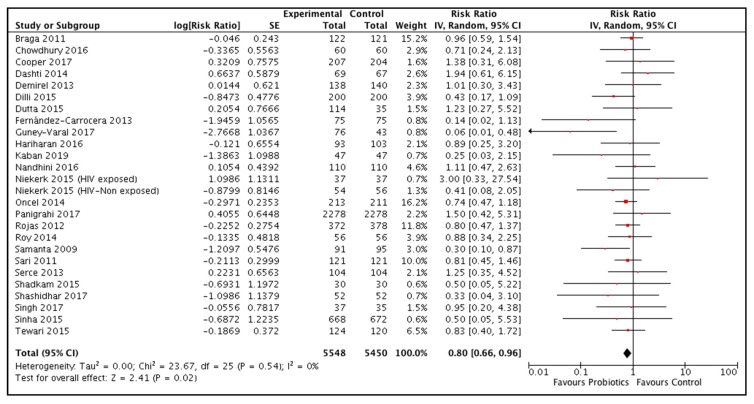
Effect of neonatal probiotic supplementation on all-cause mortality.

Twenty-five studies reported data on efficacy of probiotic supplementation during the neonatal period [18,39,40,41,43,44,45,46,47,48,49,53,54,55,56,57,58,59,61,62,63,64,65,66]. Most of the studies that reported mortality outcomes did not specify the time of deaths. So, we included the data as all-cause mortality at the longest follow up. The pooled results showed that probiotic supplementation reduced all-cause mortality by 20% compared to control (RR 0.80, 95% CI 0.66, 0.96) (Figure 3). A funnel plot for this outcome was symmetrical (Supplementary Figure S3). Subgroup analyses for this outcome are given in supplementary Table S3. The grade quality for this outcome was high (Table 2). 

##### Necrotizing Enterocolitis 

**Figure 4:**
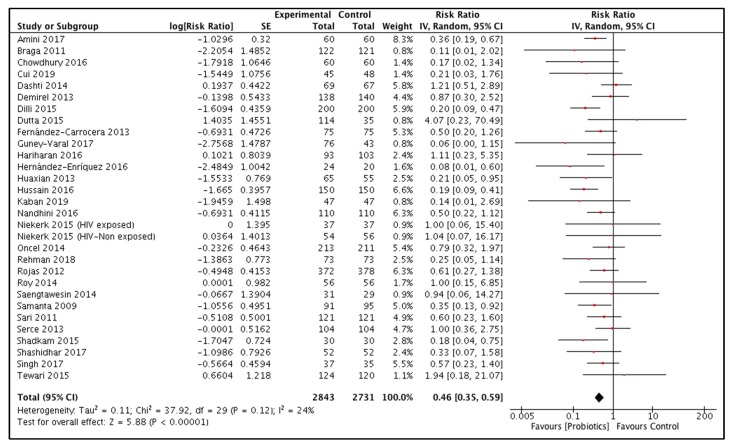
Effect of neonatal probiotic supplementation on incidence of Necrotizing Enterocolitis.

Twenty-nine studies reported this outcome and included a total of 5574 (Probiotics 2843, Control 2731) participants [18,38,39,40,42,43,44,45,46,47,48,49,50,51,52,53,54,55,56,58,59,60,61,62,63,64,66,68,69]. The pooled results showed that probiotic supplementation reduced the risk of NEC by 54% compared to the control group [RR 0.46, 95% CI 0.35, 0.59) (Figure 4). A sensitivity analysis by using fixed effect model showed the same results. A funnel plot for this outcome was symmetrical (Supplementary Figure S4). Subgroup analyses for this outcome are given in supplementary Table S3. The grade quality for this outcome was high (Table 2). 

##### Neonatal Sepsis

Data were available from twenty-one studies that included 9105 (probiotics 4606, Control 4499) [18,39,42,43,44,45,46,48,49,50,53,54,57,59,60,61,62,63,65,66,67]. The pooled results showed a reduction of 22% in the intervention group compared to control (RR 0.78, 95% CI 0.70) (Supplementary Figure S5). Subgroup analyses for this outcome was given in supplementary Table S3.

## 4. Discussion

This systematic review and meta-analysis focused on three nutrition interventions during the neonatal period i.e. vitamin A supplementation, dextrose gel supplementation and probiotic supplementation. We found 16 studies for vitamin A supplementation and 33 studies for probiotic supplementation. No study was available from low- and middle-income countries for dextrose gel supplementation during the neonatal period. Overall, there was no effect of vitamin A supplementation for all-cause mortality at 1, 6 and 12 months of age. Probiotic supplementation reduced the risk of all-cause mortality, NEC and Sepsis. 

What is the quality and applicability of these results? The data for vitamin A supplementation came from randomized trials and the included studies had minimal risk of bias. Overall number of participants was more than 150, 000. The GRADE quality assessment of the mortality outcomes was high. Thirteen of the studies were conducted in the community settings and three studies were conducted in the hospital settings. We initially planned subgroup analyses for neonatal vitamin A supplementation however we did not perform any subgroup analysis because a recent individual participant analysis (IPD) conducted subgroup analyses for 6-month mortality at individual and trial level data [21]. 

The intervention of neonatal probiotic supplementation for the prevention of morbidity and mortality in preterm/low birth weight babies seems very promising. We included 33 studies from low- and middle-income countries with an overall number of participants of 11, 595. Most of the studies had low risk of bias except three studies that were at high risk of bias for randomization/sequence generation [48,53,68]. The exclusion of these studies from meta-analyses of all-cause mortality, sepsis, and NEC did not change the results significantly. The funnel graphs for publication bias were symmetrical. The use of fixed effect models did not change the results significantly. The GRADE quality for three outcomes was high. The statistical heterogeneity in the pooled data were minimal. We, however, noted clinical heterogeneity in the use of probiotic strain, dose, and duration of supplementation. We performed a number of subgroup analyses to assess if there were any differences based on type of probiotics, single vs. multiple strain used in the formulation, use of breastmilk vs. formula milk vs. mixed. Overall, no significant difference was noted among the subgroups. 

The topic of use of probiotics for preterm/low birth weight has been debated intensely in the last decade and no consensus is reached so far. After the publication of a Cochrane review on this topic in 2011 that was later updated in 2014 [13,70], it was advocated that probiotics should be given to all the preterm/low birth weight babies admitted to the neonatal ICU. These recommendations were however challenged by other with concerns related to clinical heterogeneity of the intervention and that most of the included studies had small sample size [71,72,73]. This led to two large clinical trials, ProPrems trial and PiPS trial. The ProPrems study was conducted in Australia and New Zealand and used a mixture of probiotics (Bifidobacterium infantis, Streptococcus thermophilus, and Bifidobacterium lactis) and included 1099 preterm (less than 32 weeks) and very low birth weight (< 1500 g) neonates. The results of ProPrems trial showed that use of probiotics did not reduce the incidence of sepsis and mortality but NEC (RR 0.46, 95% CI 0.23 to 0.93) [74] compared to control. The PiPS trial included 1315 neonates between the gestational age of 23–30 weeks who were randomized within 24–48 hours to a single probiotic (Bifidobacterium breve BBG-001) or placebo. The results of PiPS trial showed no difference between the intervention and the control group for the outcomes of NEC (RR 0·93, 95% CI 0·68–1·27), sepsis (0·97, 0·73–1·29), or death (0·93, 0·67–1·30) [75]. The results of these trials were surprising in the setting of known evidence from meta-analyses. A close look at the results of the PiPS trial showed that there was significant contamination of the control group and about 49% of the neonates from the control group had same type of probiotic bacteria in their stool as those who were in the intervention group [75]. This decreased the power of the study and it was argued that a potential lack of effect might be explained by the cross-contamination of the control group [14,76]. The debate of appropriateness of probiotics for routine use in neonatal ICU has continued in the developed countries and its uptake as standard of care in neonatal ICU is variable in Europe and North America [77,78].

We included studies from low- and middle-income countries and the overall effect of probiotics for prevention of NEC, sepsis and mortality seems to hold true even when the studies from high income countries were excluded. We think that meta-analysis is appropriate to combine the included studies as the primary objective of the studies were the same i.e., to fix the dysbiosis and introduce the healthy bacteria to avoid colonization with pathogenic bacteria that could be contributing to the development of NEC and sepsis that could lead to mortality. An important consideration however from low- and middle-income countries setting is that most of the included studies were conducted in NICU settings and that facility of a NICU might not be available in the many settings in these countries. We notice a relative lack of studies from community settings. We however read the study by Panigrahi et al. with great interest [57]. This study was conducted in the community setting in rural India and included 4, 556 participants which is 3 times large than the PiPS trial. The study used a synbiotics which was a combination of a probiotic (Lactobacillus plantarum ATCC-202195) and a prebiotic (fructooligosaccharide). The study recruited neonates who were at least 35 weeks of gestation and weighed at least 2000 g. The results of the study showed a significant reduction in the primary outcome (combination of sepsis and death) in the intervention group compared to control (RR 0.60, 95% CI 0.48–0.74) and culture-positive and culture-negative sepsis and lower respiratory tract infections [57]. More such studies are needed from other countries in the community settings that include term, preterm infants and low birth weight infants. 

The strengths of this systematic review were that the review was conducted according to the standard guidelines of Campbell and Cochrane Collaboration. Two review authors screened the titles and extracted the data from selected studies. We searched multiple databases and considered studies based on a priori inclusion/exclusion criteria. Our analysis plan was predefined and we did subgroup and sensitivity analysis to assess the robustness of our results. In terms of limitations, we performed 2 post-hoc subgroup analyses for the effect of probiotic supplementation during the neonatal period. The first of this subgroup analysis was based on the type of feeding as our team thought it was essential to establish any differential effect of probiotics when the intervention was delivered with breastmilk or formula milk or both. The results for this analysis were similar among the subgroups for outcomes of all-cause mortality and NEC; however, there was significant heterogeneity among subgroups for the outcome of neonatal sepsis (Supplementary Table S3). A close examination of the data showed that the difference among subgroups was due to the group where the status of the feeding was ’unclear.’ Exclusion of this subgroup showed a homogenous protective effect in the case of breastmilk, or formula milk-fed babies against neonatal sepsis (data not shown). So we think that probiotics might have a significant protective effect against neonatal sepsis and NEC and mortality, irrespective of the type of feed offered. The second post-hoc subgroup analysis was based on settings. We were interested in knowing if the probiotics had a similar effect for infants born in the hospital compared to those in the community. We were interested in this analysis because a significant number of births happen at home in low and middle-income countries. There were a limited number of studies conducted in the community setting that addressed the effect of probiotics, so no solid conclusion could be drawn at this time for any of the outcome for these subgroups. 

## 5. Conclusions

In conclusion, vitamin A supplementation during the neonatal period in the community setting do not seem to improve all-cause mortality. No studies were available from low- and middle-income countries to assess the use of dextrose gel for prevention or treatment of hypoglycemia. Probiotics supplementation seems a very promising intervention for prevention of mortality, NEC and Sepsis in babies born low birth weight/preterm and admitted to the NICU in the low- and middle-income countries. More studies are needed in the community setting before use of probiotics can be used in the community setting in the low- and middle-income countries. 

## Figures and Tables

**Table 1:** Summary of finding table: Neonatal Vitamin A supplementation.

Outcomes	Relative Effect (95% CI)	№ of Participants (Studies)	Certainty of the Evidence (GRADE)
All-cause neonatal mortality	RR 0.99 (0.90 to 1.07)	126242 (5 RCTs)	⊕⊕⊕⊕
			HIGH
All-cause mortality at 6 months of age	RR 0.98 (0.89 to 1.08)	154940 (12 RCTs)	⊕⊕⊕⊕
			HIGH
All-cause mortality at 12 months of age	RR 1.04 (0.95 to 1.14)	118376 (8 RCTs)	⊕⊕⊕⊕
			HIGH
Adverse Events: Bulging Fontanelle 48–72 hours	RR 1.53 (1.12 to 2.09)	100562 (6 RCTs)	⊕⊕⊕⊕
			HIGH

GRADE Working Group grades of evidence; High certainty: We are very confident that the true effect lies close to that of the estimate of the effect; Moderate certainty: We are moderately confident in the effect estimate: The true effect is likely to be close to the estimate of the effect, but there is a possibility that it is substantially different; Low certainty: Our confidence in the effect estimate is limited: The true effect may be substantially different from the estimate of the effect; Very low certainty: We have very little confidence in the effect estimate: The true effect is likely to be substantially different from the estimate of effect.

**Table 2:** Summary finding table: Probiotic supplementation during the neonatal period.

	No. of Participants (Studies) Follow up	Relative Effect (95% CI)	Certainty of the Evidence (GRADE)
All-cause Mortality	10904 (25 RCTs)	RR 0.80 (0.66 to 0.96)	⊕⊕⊕⊕ HIGH ^1,2,3,4^
Neonatal Sepsis	8918 (21 RCTs)	RR 0.78 (0.70 to 0.86)	⊕⊕⊕⊕ HIGH ^1,4,5^
Necrotizing Enterocolitis	55574 (29 RCTs)	RR 0.46 (0.35 to 0.61)	⊕⊕⊕⊕ HIGH ^1,4,6^

^1^ Even though three (Fernández-Carrocera 2013; Kaban 2019; Singh 2017) of the included studies in the analysis had high risk of bias related to randomizations, the exclusion of these studies did not have much effect on the magnitude of the summary estimate or its statistical significance. ^2^ I^2^ was 0%; ^3^ All-cause mortality is an objective outcome and there were no concerns about the indirect measurement of the outcome. ^4^ The confidence interval of the summary estimate did not include 1. ^5^ I^2^ was 23% and the p value for heterogeneity was 0.16. ^6^ The I^2^ was 24%. GRADE Working Group grades of evidence; High certainty: We are very confident that the true effect lies close to that of the estimate of the effect; Moderate certainty: We are moderately confident in the effect estimate: The true effect is likely to be close to the estimate of the effect, but there is a possibility that it is substantially different; Low certainty: Our confidence in the effect estimate is limited: The true effect may be substantially different from the estimate of the effect; Very low certainty: We have very little confidence in the effect estimate: The true effect is likely to be substantially different from the estimate of effect.

## Supplementary Materials

The following are available online at https://www.mdpi.com/2072-6643/12/3/791/s1, Supplementary Document 1: Study protocol; Supplementary Document 2: Supplementary Figure S1: Risk of Bias in the include studies for neonatal vitamin A supplementation; Supplementary Figure S2: Risk of bias in the included studies for neonatal probiotic supplementation; Supplementary Figure S3: Funnel plot to show effect of probiotics for prevention of all-cause mortality during neonatal period; Supplementary Figure S4: Funnel plot to show effect of probiotics for prevention of necrotizing enterocolitis during neonatal period; Supplementary Figure S5: Effect of neonatal probiotics for prevention of incidence of neonatal sepsis; Supplementary table S1: Characteristics of included for vitamin A supplementation; Supplementary table S2: Characteristic of included studies: Probiotic supplementation during neonatal period. Supplementary table S3: Subgroup analyses for probiotic supplementation during neonatal period; Supplementary Table S4: Table of excluded studies

## Appendix A

Search Strategies

Medline Strategy using PubMed

**Vitamin A**

((((("Vitamin A"[Mesh]) OR (Vitamin A[tiab] OR Aquasol A[tiab] OR Retinol[tiab] OR All Trans Retinol[tiab] OR All-Trans-Retinol[tiab] OR Vitamin A1[tiab] OR Vitamin A 1[tiab] OR 11-cis-Retinol[tiab] OR 11 cis Retinol[tiab] OR Tretinoin[tiab]) AND Supplement*[tiab])) AND (("Infant"[Mesh] OR "Premature Birth"[Mesh]) OR (Neonat*[tiab] OR neo nat*[tiab]) OR (newborn* OR new Born*[tiab] OR newly born*[tiab]) OR (preterm[tiab] OR preterms[tiab] OR pre term[tiab] OR pre terms[tiab]) OR (premature*[tiab] AND (birth*[tiab] OR born[tiab] OR deliver*[tiab])) OR (low[tiab] AND (birthweight*[tiab] OR birth weight*[tiab])) OR (lbw[tiab] OR vlbw[tiab] OR elbw[tiab]) OR infant*[tiab] OR (baby[tiab] OR babies[tiab])))) NOT ("Animals"[Mesh] NOT ("Animals"[Mesh] AND "Humans"[Mesh]))

**Glucose**

(((((("Glucose"[Mesh]) OR (Dextrose OR Glucose[tiab]) AND supplement*))) AND (("Infant"[Mesh] OR "Premature Birth"[Mesh]) OR (Neonat*[tiab] OR neo nat*[tiab]) OR (newborn* OR new Born*[tiab] OR newly born*[tiab]) OR (preterm[tiab] OR preterms[tiab] OR pre term[tiab] OR pre terms[tiab]) OR (premature*[tiab] AND (birth*[tiab] OR born[tiab] OR deliver*[tiab])) OR (low[tiab] AND (birthweight*[tiab] OR birth weight*[tiab])) OR (lbw[tiab] OR vlbw[tiab] OR elbw[tiab]) OR infant*[tiab] OR (baby[tiab] OR babies[tiab])))) NOT ("Animals"[Mesh] NOT ("Animals"[Mesh] AND "Humans"[Mesh]))

**Probiotics**

((((("Probiotics"[Mesh] OR "Prebiotics"[Mesh] OR "Synbiotics"[Mesh]) OR (Probiotic*[tiab] OR prebiotic*[tiab] OR synbiotic*[tiab]))) AND (("Infant"[Mesh] OR "Premature Birth"[Mesh]) OR (Neonat*[tiab] OR neo nat*[tiab]) OR (newborn* OR new Born*[tiab] OR newly born*[tiab]) OR (preterm[tiab] OR preterms[tiab] OR pre term[tiab] OR pre terms[tiab]) OR (premature*[tiab] AND (birth*[tiab] OR born[tiab] OR deliver*[tiab])) OR (low[tiab] AND (birthweight*[tiab] OR birth weight*[tiab])) OR (lbw[tiab] OR vlbw[tiab] OR elbw[tiab]) OR infant*[tiab] OR (baby[tiab] OR babies[tiab])))) NOT ("Animals"[Mesh] NOT ("Animals"[Mesh] AND "Humans"[Mesh]))

**CINAHL Strategies**

**Vitamin A**

( MH ”Vitamin A") OR TI ( "Vitamin A" OR "Aquasol A" OR Retinol OR "All Trans Retinol" OR "All-Trans-Retinol" OR "Vitamin A1" OR "Vitamin A 1" OR "11-cis-Retinol" OR "11 cis Retinol" OR Tretinoin ) OR AB ( "Vitamin A" OR "Aquasol A" OR Retinol OR "All Trans Retinol" OR "All-Trans-Retinol" OR "Vitamin A1" OR "Vitamin A 1" OR "11-cis-Retinol" OR "11 cis retinol" OR Tretinoin )

AND

TI ( Supplement* ) OR AB (Supplement* ) OR MH "Dietary Supplementation" OR MH "Dietary Supplements"

AND

(MH "Infant" OR MH "Infant, Premature" OR MH "Infant, Newborn") OR TI ( (Neonat* OR neo nat*) OR (newborn* OR new Born* OR newly born*) OR (preterm OR preterms OR pre term OR pre terms) OR (premature* AND (birth* OR born OR deliver*)) OR (low AND (birthweight* OR birth weight*)) OR (lbw OR vlbw OR elbw) OR infant* OR (baby OR babies) ) OR AB ( (Neonat* OR neo nat*) OR (newborn* OR new Born* OR newly born*) OR (preterm OR preterms OR pre term OR pre terms) OR (premature* AND (birth* OR born OR deliver*)) OR (low AND (birthweight* OR birth weight*)) OR (lbw OR vlbw OR elbw) OR infant* OR (baby OR babies) )

NOT

(MH "Animals" NOT (MH "Animals" AND MH "Humans"))

**Limiter: Exclude MEDLINE records**

**Glucose**

(MH "Glucose") OR TI ( Dextrose OR Glucose ) OR AB ( Dextrose OR Glucose )

AND

TI ( Supplement* ) OR AB (Supplement* ) OR MH "Dietary Supplementation" OR MH "Dietary Supplements"

AND

(MH "Infant" OR MH "Infant, Premature" OR MH "Infant, Newborn") OR TI ( (Neonat* OR neo nat*) OR (newborn* OR new Born* OR newly born*) OR (preterm OR preterms OR pre term OR pre terms) OR (premature* AND (birth* OR born OR deliver*)) OR (low AND (birthweight* OR birth weight*)) OR (lbw OR vlbw OR elbw) OR infant* OR (baby OR babies) ) OR AB ( (Neonat* OR neo nat*) OR (newborn* OR new Born* OR newly born*) OR (preterm OR preterms OR pre term OR pre terms) OR (premature* AND (birth* OR born OR deliver*)) OR (low AND (birthweight* OR birth weight*)) OR (lbw OR vlbw OR elbw) OR infant* OR (baby OR babies) )

NOT

(MH "Animals" NOT (MH "Animals" AND MH "Humans"))

**Limiter: Exclude MEDLINE records**

**Probiotics**

(MH "Probiotics") OR (MH "Prebiotics") OR TI ( probiotic* OR prebiotic* OR synbiotic* ) OR AB ( probiotic* OR prebiotic* OR synbiotic* )

AND

(MH "Infant" OR MH "Infant, Premature" OR MH "Infant, Newborn") OR TI ( (Neonat* OR neo nat*) OR (newborn* OR new Born* OR newly born*) OR (preterm OR preterms OR pre term OR pre terms) OR (premature* AND (birth* OR born OR deliver*)) OR (low AND (birthweight* OR birth weight*)) OR (lbw OR vlbw OR elbw) OR infant* OR (baby OR babies) ) OR AB ( (Neonat* OR neo nat*) OR (newborn* OR new Born* OR newly born*) OR (preterm OR preterms OR pre term OR pre terms) OR (premature* AND (birth* OR born OR deliver*)) OR (low AND (birthweight* OR birth weight*)) OR (lbw OR vlbw OR elbw) OR infant* OR (baby OR babies) )

NOT

(MH "Animals" NOT (MH "Animals" AND MH "Humans"))

Limiter: Exclude MEDLINE records

**Scopus Strategies**

**Vitamin A**

(TITLE-ABS("Vitamin A" OR "Aquasol A" OR retinol OR "All Trans Retinol" OR "Vitamin A1" OR "11-cis-Retinol" OR tretinoin)) AND (TITLE-ABS(Supplement*)) AND (TITLE-ABS ( ( neonat* OR "neo nat*" ) OR ( newborn* OR "new born*" OR "newly born*" ) OR ( preterm OR preterms OR "pre term" OR "pre terms" ) OR ( premature* ) AND ( birth* OR born OR deliver* ) OR ( low AND ( birthweight* OR "birth weight*" ) ) OR ( lbw OR vlbw OR elbow ) OR infant* OR ( baby OR babies ) )) AND NOT INDEX(medline)

**Glucose**

TITLE-ABS (Glucose OR Dextrose) AND TITLE-ABS (supplement*) AND TITLE-ABS ( ( neonat* OR "neo nat*" ) OR ( newborn* OR "new born*" OR "newly born*" ) OR ( preterm OR preterms OR "pre term" OR "pre terms" ) OR ( premature* ) AND ( birth* OR born OR deliver* ) OR ( low AND ( birthweight* OR "birth weight*" ) ) OR ( lbw OR vlbw OR elbow ) OR infant* OR ( baby OR babies ) ) AND NOT INDEX ( medline )

**Probiotics**

TITLE-ABS (Probiotic* OR Prebiotic* OR Synbiotic*) AND TITLE-ABS (( neonat* OR "neo nat*" ) OR ( newborn* OR "new born*" OR "newly born*" ) OR ( preterm OR preterms OR "pre term" OR "pre terms" ) OR ( premature* ) AND ( birth* OR born OR deliver* ) OR ( low AND ( birthweight* OR "birth weight*" ) ) OR ( lbw OR vlbw OR elbow ) OR infant* OR ( baby OR babies ) ) AND NOT INDEX ( medline )

**CENTRAL**

**Vitamin A**

1 MeSH descriptor: [infant] explode all trees

2 MeSH descriptor: [Premature Birth] explode all trees

3 (Neonat*:ti,ab OR neo nat*:ti,ab) OR (newborn*:ti,ab OR new Born*:ti,ab OR newly born*:ti,ab) OR (preterm:ti,ab OR preterms:ti,ab OR pre term:ti,ab OR pre terms:ti,ab) OR (premature*:ti,ab AND (birth*:ti,ab OR born:ti,ab OR deliver*:ti,ab)) OR (low:ti,ab AND (birthweight*:ti,ab OR birth weight*:ti,ab)) OR (lbw:ti,ab OR vlbw:ti,ab OR elbw:ti,ab) OR infant*:ti,ab OR (baby:ti,ab OR babies:ti,ab)

4 #1 OR #2 OR #3

5 MeSH descriptor: [Animals] explode all trees

6 MeSH descriptor: [Humans] explode all trees

7 (#5 NOT (#5 AND #6))

8 supplement*:ti,ab

9 MeSH descriptor: [Vitamin A] explode all trees

10 "Vitamin A":ti,ab OR "Aquasol A":ti,ab OR Retinol:ti,ab OR "All Trans Retinol":ti,ab OR "All-Trans-Retinol":ti,ab OR "Vitamin A1":ti,ab OR "Vitamin A 1":ti,ab OR "11 cis Retinol":ti,ab OR "11-cis-Retinol":ti,ab OR Tretinoin:ti,ab

11 #9 OR #10

12 #11 AND #8

13 #12 AND #4 NOT #7

14 "accession number" near pubmed

15 #13 NOT #14

**Glucose**

1 MeSH descriptor: [infant] explode all trees

2 MeSH descriptor: [Premature Birth] explode all trees

3 (Neonat*:ti,ab OR neo nat*:ti,ab) OR (newborn*:ti,ab OR new Born*:ti,ab OR newly born*:ti,ab) OR (preterm:ti,ab OR preterms:ti,ab OR pre term:ti,ab OR pre terms:ti,ab) OR (premature*:ti,ab AND (birth*:ti,ab OR born:ti,ab OR deliver*:ti,ab)) OR (low:ti,ab AND (birthweight*:ti,ab OR birth weight*:ti,ab)) OR (lbw:ti,ab OR vlbw:ti,ab OR elbw:ti,ab) OR infant*:ti,ab OR (baby:ti,ab OR babies:ti,ab)

4 #1 OR #2 OR #3

5 MeSH descriptor: [Animals] explode all trees

6 MeSH descriptor: [Humans] explode all trees

7 (#5 NOT (#5 AND #6))

8 supplement*:ti,ab

9 MeSH descriptor: [Glucose] explode all trees

10 Dextrose:ti,ab OR Glucose:ti,ab

11 #9 OR #10

12 #11 AND #8

13 #12 AND #4 NOT #7

14 "accession number" near pubmed

15 #13 NOT #14

**Probiotics**

1 MeSH descriptor: [infant] explode all trees

2 MeSH descriptor: [Premature Birth] explode all trees

3 (Neonat*:ti,ab OR neo nat*:ti,ab) OR (newborn*:ti,ab OR new Born*:ti,ab OR newly born*:ti,ab) OR (preterm:ti,ab OR preterms:ti,ab OR pre term:ti,ab OR pre terms:ti,ab) OR (premature*:ti,ab AND (birth*:ti,ab OR born:ti,ab OR deliver*:ti,ab)) OR (low:ti,ab AND (birthweight*:ti,ab OR birth weight*:ti,ab)) OR (lbw:ti,ab OR vlbw:ti,ab OR elbw:ti,ab) OR infant*:ti,ab OR (baby:ti,ab OR babies:ti,ab)

4 #1 OR #2 OR #3

5 MeSH descriptor: [Animals] explode all trees

6 MeSH descriptor: [Humans] explode all trees

7 (#5 NOT (#5 AND #6))

8 MeSH descriptor: [Probiotics] explode all trees

9 MeSH descriptor: [Prebiotics] explode all trees

10 MeSH descriptor: [Synbiotics] explode all trees

11 #8 OR #9 OR #10

12 Probiotic*:ti,ab OR prebiotic*:ti,ab OR synbiotic*:ti,ab

13 #11 or #12

14 #13 AND #4 NOT #7

15 "accession number" near pubmed

16 #14 NOT #15

**LILACS**

**Vitamin A**

(tw:(( "Vitamin A" ) )) OR (ti:(("Aquasol A" OR retinol OR "All Trans Retinol" OR "Vitamin A1" OR "11-cis-Retinol" OR tretinoin ) )) OR (ab:(("Aquasol A" OR retinol OR "All Trans Retinol" OR "Vitamin A1" OR "11-cis-Retinol" OR tretinoin ) )) AND (ti:(supplement*)) OR (ab:(supplement*)) AND (tw:(Infant)) OR (tw:("Premature Birth")) OR (ti:(( ( neonat* OR "neo nat*" ) OR ( newborn* OR "new born*" OR "newly born*" ) OR ( preterm OR preterms OR "pre term" OR "pre terms" ) OR ( premature* ) AND ( born OR deliver* ) OR ( low AND ( birthweight* OR "birth weight*" ) ) OR ( lbw OR vlbw OR elbw ) OR ( baby OR babies ) ))) OR (ab:(( ( neonat* OR "neo nat*" ) OR ( newborn* OR "new born*" OR "newly born*" ) OR ( preterm OR preterms OR "pre term" OR "pre terms" ) OR ( premature* ) AND ( born OR deliver* ) OR ( low AND ( birthweight* OR "birth weight*" ) ) OR ( lbw OR vlbw OR elbw ) OR ( baby OR babies ) ))) AND db:("LILACS")

**Glucose**

((tw:(glucose)) OR (ti:(dextrose)) OR (ab:(dextrose)) AND (ti:(supplement*)) OR (ab:(supplement*))) AND ((tw:(infant )) OR (tw:(“premature birth”)) OR (ti:(( neonat* OR "neo nat*" ) OR ( newborn* OR "new born*" OR "newly born*" ) OR ( preterm OR preterms OR "pre term" OR "pre terms" ) OR ( premature* ) )) AND (ti:(( born OR deliver* ) OR ( low AND ( birthweight* OR "birth weight*" ) ) OR ( lbw OR vlbw OR elbw ) OR ( baby OR babies ))) OR (ab:(( neonat* OR "neo nat*" ) OR ( newborn* OR "new born*" OR "newly born*" ) OR ( preterm OR preterms OR "pre term" OR "pre terms" ) OR ( premature* ) )) AND (ab:(( born OR deliver* ) OR ( low AND ( birthweight* OR "birth weight*" ) ) OR ( lbw OR vlbw OR elbw ) OR ( baby OR babies )))) AND (instance:"regional") AND ( db:("LILACS"))

**Probiotics**

((tw:(probiotics OR prebiotics OR synbiotics)) OR (ti:(probiotic* OR prebiotic* OR synbiotic*)) OR (ab:(probiotic* OR prebiotic* OR synbiotic*))) AND ((tw:(infant )) OR (tw:(“premature birth”)) OR (ti:(( neonat* OR "neo nat*" ) OR ( newborn* OR "new born*" OR "newly born*" ) OR ( preterm OR preterms OR "pre term" OR "pre terms" ) OR ( premature* ) )) AND (ti:(( born OR deliver* ) OR ( low AND ( birthweight* OR "birth weight*" ) ) OR ( lbw OR vlbw OR elbw ) OR ( baby OR babies ))) OR (ab:(( neonat* OR "neo nat*" ) OR ( newborn* OR "new born*" OR "newly born*" ) OR ( preterm OR preterms OR "pre term" OR "pre terms" ) OR ( premature* ) )) AND (ab:(( born OR deliver* ) OR ( low AND ( birthweight* OR "birth weight*" ) ) OR ( lbw OR vlbw OR elbw ) OR ( baby OR babies )))) AND (instance:"regional") AND ( db:("LILACS"))

**EMBASE**

**Vitamin A**

1 ’retinol’/exp OR ’retinol palmitate’/exp OR ’11 cis retinol’/exp OR ’retinoic acid’/exp

2 ’vitamin a’:ti,ab OR ’aquasol a’:ti,ab OR retinol:ti,ab OR ’all trans retinol’:ti,ab OR ’all-trans-retinol’:ti,ab OR ’vitamin a1’:ti,ab OR ’vitamin a 1’:ti,ab OR ’11-cis-retinol’:ti,ab OR ’11 cis retinol’:ti,ab OR tretinoin:ti,ab

3 supplement*:ti,ab

4 ’supplementation’/exp

5 #1 OR #2

6 #3 OR #4

7 #5 AND #6

8 ’infant’/exp OR ’prematurity’/exp OR ’newborn’/exp OR ’low birth weight’/exp OR ’very low birth weight’/exp OR ’extremely low birth weight’/exp OR ’premature labor’/exp

9 neonat*:ti,ab OR ’neo nat*’:ti,ab OR newborn*:ti,ab OR ’new born*’:ti,ab OR ’newly born*’:ti,ab OR preterm:ti,ab OR preterms:ti,ab OR ’pre term’:ti,ab OR ’pre terms’:ti,ab OR (premature*:ti,ab AND (birth*:ti,ab OR born:ti,ab OR deliver*:ti,ab)) OR (low:ti,ab AND (birthweight*:ti,ab OR ’birth weight*’:ti,ab)) OR lbw:ti,ab OR vlbw:ti,ab OR elbw:ti,ab OR infant*:ti,ab OR baby:ti,ab OR babies:ti,ab

10 #8 OR #9

11 #7 AND #10

12 #11 NOT ([animals]/lim NOT [humans]/lim)

13 #12 NOT [medline]/lim
