# Supplementary material for: Effect of Synthetic Vitamin A and Probiotics Supplementation for Prevention of Morbidity and Mortality during the Neonatal Period. A Systematic Review and Meta-Analysis of Studies from Low- and Middle-Income Countries
Source: Nutrients. 2020 Mar 17;12(3):791. doi: 10.3390/nu12030791 (PMC7146603; doi:10.3390/nu12030791)
Supplement: Supplementary file 1 [file nutrients-12-00791-s001.zip › Supplementary Document 2- Supplementary Tables and Figures.docx]

Supplementary Document 2:

Figure S1: Risk of Bias in the include studies for neonatal vitamin A supplementation


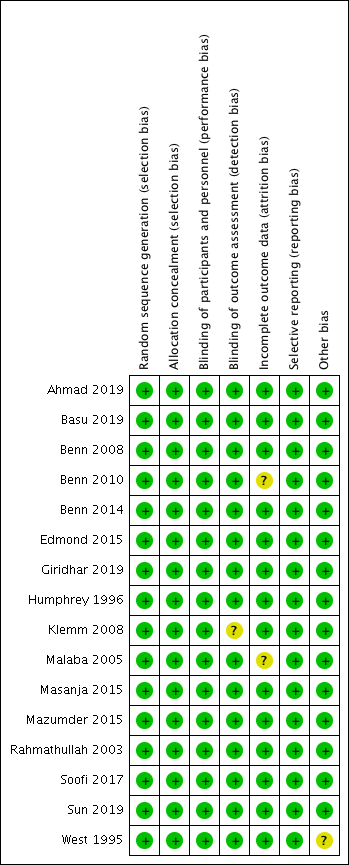


Figure S2: Risk of bias in the included studies for neonatal probiotic supplementation


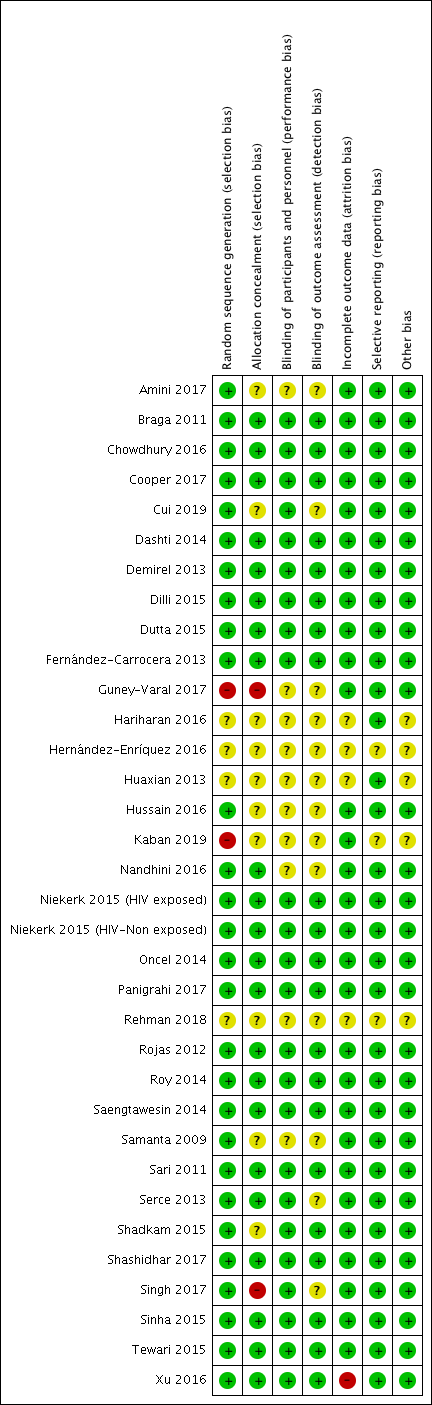


Supplementary Figure S3: Funnel plot to show effect of probiotics for prevention of all-cause mortality during neonatal period.


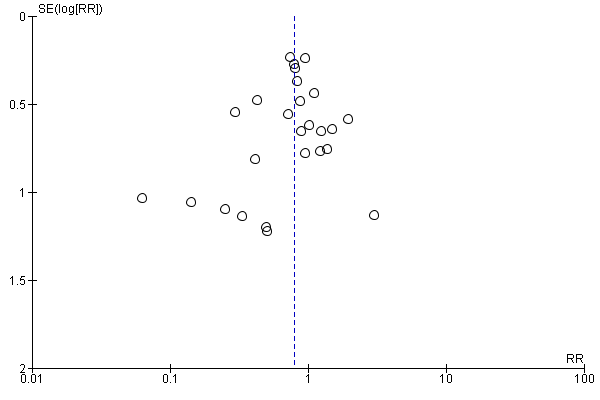


Supplementary figure S4: Funnel plot to show effect of probiotics for prevention of necrotizing enterocolitis during neonatal period.


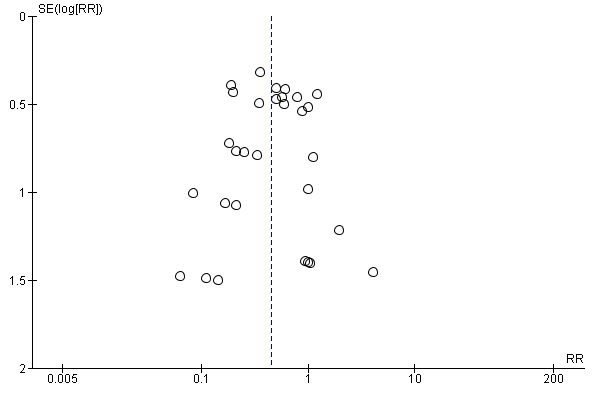


Supplementary Figure S5: Effect of neonatal probiotics for prevention of incidence of neonatal sepsis


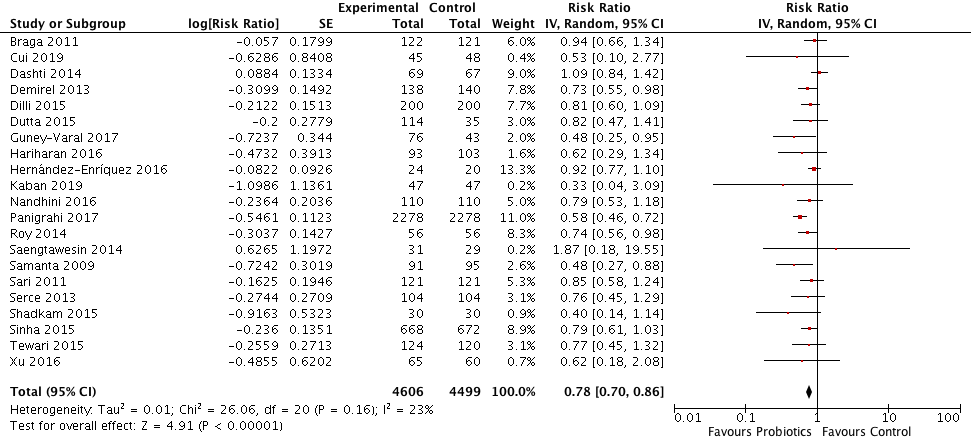


Supplementary table S1: Characteristics of included for vitamin A supplementation

| Study | Country | Study Design | Description of Participants (Give inclusion/Exclusion Criteria) | % Female (N) | Number of Participants in Intervention Group | Number of Participants in Control Group | Total Duration of Intervention | Comparison? (E.g. placebo, etc.) |
| --- | --- | --- | --- | --- | --- | --- | --- | --- |
| Klemm RD 2008 | Bangladesh | Double-masked cluster-randomized placebo-controlled trial | Infants born to consenting mothers participating in the parent trial/infants who have died prior reciving vitamin a supplementation, those born outside studies area and those that could not be reached during first 30 days after birth | 49% (7800) | 7,953 | 7984 | 72 hours | Placebo |
| Malaba LC 2005 | Zimbabwe | randomized, placebo-controlled, 2-by-2 factorial design trial | Participants were eligible if they had no acutely life-threatning condition, infant was a singleton with birth weight 1500g,andthemotherplanned to stay in Harare after delivery | 48% (4436) | 4599 | 4609 | 72 hours | Placebo |
| Masanja H 2005 | Tanzania | Randomized double-blind, placebo-controlled trial | Newborns who were able to feed orally, the family intended to stay in the study area for at least 6 months, and if parents provided written informed consent to participate. Babies who were enrolled in other trials were excluded. | 47.6% (15216) | 15995 | 16004 | 72 hours | Placebo |
| Mazumder S 2015 | India | Randomized double-blind, placebo-controlled trial | Eligible participants were neonates whose parents consented to participate, were likely to stay in the study area until at least 6 months of age, and were able to feed orally at the time of enrolment/Newborn babies who were unable to feed or whose feeding could not be assessed, or whose family did not intend to stay in the study area for at least 6 months, were excluded. | 47.9% (21566) | 22493 | 22491 | 72 hours | Placebo |
| Rahmathulla 2003 | India | Randomized, placebo controlled, community based trial | Liveborn infants that resulted from all pregnancies within participating villages were eligible for participation. | 48.6% (5651) | 5786 | 5833 | 72 hours | Placebo |
| West 2003 | Nepal | Randomized,double-masked, placebo-controlled community trial | Elligible participants included infants less than 6 months old/ Infants 6 mo of age were excluded. | 49.4% (5890) | 6082 | 5832 | 72 hours | Placebo |
| Benn 2008 | Guinea-Bissau | Randomized placebo controlled trial | Elligible participants included neonates who weigh at least 2500 g at presentation and no signs of overt illness or malformations. | 49.4% (2145) | 2145 | 2200 | 72 hours | Placebo |
| Benn 2010 | Guinea-Bissau | Randomized, placebo, controlled, two by two factorial trial | Elligible participants included neonates who weigh less than 2500 g at presentation and had no signs of severe illness or malformations. | 55.8% (959) | 854 | 863 | 72 hours | Placebo |
| Benn 2014 | Guinea-Bissau | Randomized, double-blind, placebo-controlled trial | Eligible participants were healthy normal-birth-weight neonates who were due to be administered BCG vaccine | 49% (2943) | 4026 | 2022 | 72 hours | Placebo |
| Sufi 2016 | Pakistan | Community-based, placebo-controlled, cluster randomized trial | All live born infants within participating villages were potentially eligible for inclusion in the study. Infants with obvious congenital malformations and birth weight <1500 g were exclud | 48% (5286) | 5645 | 5378 | 72 hours | Placebo |
| Edmond 2015 | Ghana | Randomized, double-blind, placebo-controlled trial | The trial participants were at least 2 h old, identiﬁ ed at home or facilities on the day of birth or in the next 2 days, able to feed orally, likely to stay in the study area for at least 6 months, and at least one parent provided written informed consent for participation. | 49% (11306) | 11474 | 11481 | 72 hours | Placebo |
| Humphrey 1996 | Indonesia | Placebo-controlled trial | All infants bom at Hasan Sadikin Hospital in Bandung, Indonesia, with weights greater than 1500g and no signs of life-threatening illness. Very low birth weight infants (<1500 gin) and those with life-threatening illness were excluded. | 48% (992) | 1034 | 1033 | 72 hours | Placebo |
| Basu 2019 | India | Randomized, double-blind, placebo controlled trial | Inclusion: Inborn,VLBW(birthweight(BW)<1500g) neonates admitted in NICU and requiring respiratory support in the form of oxygen inhalation through nasal prongs or head box, CPAP, HFNC,or mechanical ventilation at the age of 24h Exclusion:Neonates with major congenital malformation, any life-threatening condition where immediate oral feeding was contraindicated such as reversal of umbilical artery enddiastolic blood flow on antenatal Doppler, perinatal asphyxia with moderate to severe hypoxic ischemic encephalopathy, shock with escalating doses of vasopressors, recurrent seizures, and suspected inborn errors of metabolism. | 43.9% (86) | 98 | 98 | Every other day | Placebo |
| Sun 2019 | China | Randomized control trial | Inclusion: infants admitted to the neonatal intensive care unit at a gestational age of ,28 weeks, ,96 hours of age. Exclusion: Infants with genetic metabolic diseases; congenital major abnormalities; congenital TORCH infections with overt signs at birth; terminal stage of illness (pH ,7.0 or hypoxia with bradycardia .2 hours); or the lack of parental consent | 35.1% (92) | 132 | 130 | Daily | Placebo (Soybean Oil) |
| Ahmed 2019 | Bangladesh | RCT | Inclusion criteria: "consent of the mother and willingness to have their infant participate; singleton birth at MCHTI clinic and eligible for vaccination according to the national and MCHTI clinic policy".  Exclusion criteria: "planned at home delivery because of the low likelihood of vaccination at MCHTI within 48 h of birth, (2) congenital disease or a serious infection showing that the infant was not healthy; infant with birth weight less than 1500 g and inability to enroll within 48 h of birth due to lack of timely notification or other exceptional circumstances". | 50% | 153 | 153 | Single dose | Placebo (Soybean Oil) |
| Girhidar 2019 | India | RCT | Inclusion criteria: All infants admitted to the neonatal intensive care unit with birth weight between 750 and 1250 grams and between 24 to 96 hours of life  Exclusion criteria: lethal congenital malformations, terminal illness characterized by shock or bradycardia for more than 2 hours, refusal of consent | 40 | 61 | 59 | 28 days | Placebo Normal saline |

Supplementary table S2: Characteristic of included studies: Probiotic supplementation during neonatal period

| Study | Country | Study type | Description of Participants (Inclusion/exclusion Criteria) | % Female (N) (Give for total study population) | Number of Participants in Intervention Group | Number of Participants in Control Group | Intervention Form (E.g. tablet, drop, syrup) | Total Duration of Intervention | Frequency of Intervention (E.g. daily, weekly, etc.) | Dose | Strain of Probiotic | Comparison? (E.g. placebo, etc.) |
| --- | --- | --- | --- | --- | --- | --- | --- | --- | --- | --- | --- | --- |
|  |  |  |  |  |  |  |  |  |  |  |  |  |
| Amini 2017 | Iran | Randomized clinical trial | All premature newborns (n = 115) weighting 750-1500 g or <32 weeks gestation who received antibiotics and total parenteral nutrition in NICU of Vali Asr Hospital were included. Premature babies less than 750 and more than 1500 grams and neonateswithcongenitalheartdisease,congenitalmalformations,andimmunesystemdeﬁciency,evenintheirfamily members, were excluded |  | 60 | 55 | Powder | 13 days | 8-10 times daily | 0.8-1g per day | Strep thermophilus, lactobacillus rhamnosus, lactobacillus acidophilus, lactobacilus bulgaricus, bifidobacterium infantis, lactobacillus casei | Enteral feed without probiotic |
| Braga 2011 | Brazil | RCT | unclear, but "no major congenital malformations, life threatening chromosomal alterations, or congenital infections" | 51.5% (119) from I: 51.26% (61), C: 51.76% (58) | 119 | 112 | powder in 3mL human banked milk | day 2 of life to day 30 of life, NEC, discharge, or death | daily | 3.5 x 10*7 L. casei and 3.5x10*9 cfu B. breve | L. casei and B. breve | 3mL banked milk without probiotics |
| Chowdhury 2016 | Bangladesh | Randomized control | Preterm (<33 wks), VLBW (<1500gms) infants who are able to tolerate oral feeds and survive beyond 48 hrs. Babies with suspicion of clinical sepsis, presence of prenatal asphyxia, major congenital anomaly and babies who expired due to other neonatal illness were excluded | 32.4% (33) | 60 | 60 | Powder | 10 days | daily | 3x10^9 CFU/day | Bifidobactrium spp, lactobacillus | Breastmilk without probiotic |
| Cooper 2016 | South Africa | multicenter RCT | "healthy", full term (37-42 wks), HIV+ formula feeding mothers, <=3 days old, 2500-4500g, singleton birth; E: congenital illness or malformation affecting growth; significant perinatal disease, antibiotics in 1st 3 days of life, caregivers could not comply, or in another trial | 47.98 (202) | 207 | 214 | powder mixed into formula | enrollment to 6 months of age then received pro/prebiotic free formula | multiple times daily with every bottle/feeding | galactooligosaccharides an dmilk oligosaccharides total 5.8 +-1 g/100g of powder fomrula (8g/L liquid formula) + B. lactis CNCMI3446 1x10*7 cfu/g powder formula | b. lactis | placebo: formula without pre and probiotics |
| Cui 2019 | China | prospective, double-blinded randomized study | quote: "Inclusion criteria: formula-fed preterm infants admitted within 12 h of birth whose gestational age ≥ 30 and < 37 weeks; birthweight≥1500 g and ≤ 2000 g with vital sign  and hemodynamic parameters stable. Exclusion cri- teria: congenital diseases, expected hospitalization less  than 2 weeks and maternal or neonatal antibiotics or other probiotics before admission." | 51.61% | 45 | 48 | oral drops | minimum of 7 days | 5 drops daily | 1 × 10^8 colony-forming units | L. reuteri | placebo |
| Demirel 2013 | Turkey | prospective, blinded, rct | I: <=32 weeks and birthweight <=1500g who survived to start enteral feedings. E: major congenital anomalies, lack of parental consent, death in first seven days after study start | 50.2% (136) | 135 | 136 | mixed into breast milk or formula | until discharge | once a day | 250mg (5 billion cfu) | S boulardii | no addition to breastmilk/ formula |
| Dashti 2014 | Iran | prospective triple-blinded, interventional, randomized clinical trial | Quote: "Inclusion criteria were: birth weight of 700-1800 g, stable hemodynamic, be able to have enteral feeding, and written parental consent, exclusion criteria were: evidence or suspicion of congenital intestinal obstruction or perforation, prenatal or postnatal diagnosis of gastroschisis, large omphalocele, or congenital diaphragmatic hernia, and major congenital anomalies." | 45.60% | 69 | 67 | standard milk supplemented with once a day probiotics supplement |  | once daily | quote: "Neonates weighing less than 1000 g were fed with a half of sachet once daily (5 × 108 CFU of probiotics), neonates weighing 1001–1500g were fed with 3/4 of a sachet once daily (7.5 × 108 CFU of probiotics), neonates weighing more than 1500 g were fed with a full sachet once daily (1 × 109 CFU of probiotics)." | Lactobacillus acidophilus, Lactobacillus rhamnosus, Bifidobacterium longum, Lactobacillus bulgaricus, Lactobacillus casei, Streptococcus thermophilus, Bifidobacterium breve, and Bifidobacterium | placebo |
| Dilli 2015 | Turkey | randomized, controlled trial | quote: "VLBW infants with a gestational age of <32 weeks and a birth weight of <1500 g, born at or transferred to the NICU within the first week of life and fed enterally before inclusion, were eligible. Infants with any disease other than those linked to prematurity or congenital anomalies of the intestinal tract, not fed enterally or who died before the sev- enth day after birth, whose mothers had taken nondietary  probiotic supplements, and whose parents refused to partic- ipate were excluded." | 46% | 300 | 100 | 1 sachet per day with breast milk or formula | 8 weeks or until death | once daily | 30 mg with 1 ml of sterile water or breast milk | probiotic (Bifidobacterium lactis, 5 109 colony-forming units), prebiotic (inulin, 900 mg), or synbiotic (Bifidobacterium lactis, 5 109 colony-forming units,) | placebo |
| Dutta 2015 | India | randomized, controlled trial | Inclusion Criteria:  (1) Neonates born at 27 to 33 weeks gestation in our hospital  (2) aged less than 96 hours of life  (3) who were likely to either remain admitted in hospital or reside within 30 km of the hospital for the next 28 days  (4) who were tolerating at least 15 ml/kg/day of milk feeds.  Exclusion Criteria:  (1) a gastro-intestinal malformation  (2) prior NEC or sepsis  (3) any life-threatening malformation that limited estimated life expectancy to less than a month | 35% | 114 | 38 | Contents of the probiotic sachet were mixed with milk in steel containers with thorough aseptic precautions and immediately administered. The route of administration was a feeding tube in those who were gavage fed (followed by a sterile water flush to clear the feeding tube) or by a sterile spoon in those taking oral feeds. | 21 days | Once daily | 3 groups with different doses | . The probiotic sachets with a dose of 10 billion contained a combination of Lactobacillus acidophilus (5,300 million), Lactobacillus rhamnosus (2,900 million), Bifidobacterium longum (700 million), and Saccharomyces boulardii (1,100 million). | Placebo |
| Fernandez 2012 | Mexico | prospective rct double blind | I: <1500g, preterm; E: <1500g with low apgar <6 at 5min, GI malformations, genetic syndromes, asphyxia and IA-IB NEC (bell's) | NA | 75 | 75 | powder as suspension (1g packet in 3mL of breastmlk or preterm formula) | 3-114 days per table 2, definition NA, maybe start of enteral feedings until discharge including any paused days for enteral intolerance? | daily | L. acidophilus 1x10*9 cfu/g,, L rhamnosus 4.4x10*8 cfu/g, L casei 1x10*9 cfu/g, L plantarum 1.76x10*8 cfu/g, Bifido infantis 2.76x10*7 cfu/g, Streptococcus thermophilus 6.6x10*5 cfu/g | L. acidophilus, L rhamnosus, L casei, L plantarum, Bifido infantis, Streptococcus thermophilus | BM or formula without probiotics (placebo) |
| Güney-Varal 2017 |  | Prospective, randomized control trial | Preterm infants with a gestational age ≤32 week and a birth weight ≤ 1500 g at Uludağ University Medical Faculty Neonatal Intensive Care Unit (NICU)... The infants  with: detected chromosomal abnormalities, previous gastrointestinal system surgery, a diagnosis of metabolic disease, babies lost in the first postnatal week and babies with severe sepsis episode were excluded from the study. | 42% | 76 | 43 | Administered orally during feeding | Until discharge from NICU | Daily |  | Lactobacillus rhamnosus, Lactobacillus casei, Lactobacillus plantorum, & Bifidobacterium animalis | Placebo: nonsupplemented breast milk |
| Hariharaan 2016 | 960 | NA | Inclusion Criteria: "Infants with birth weight < 1250g, gestation <32 weeks" | NA | 93 | 103 | twice daily | 6 weeks | twice daily | 2.5 X 109 UFC of each twice a day, from the 3rd day of life, for 6 week courses | Intervention: Lactobacillus acidophilus, Bifidobacterium bifidum, Saccharomyces boulardii 2.5 X 109 UFC of each twice a day, from the 3rd day of life, for 6 week courses | No probiotics |
| Hernandez-Enriquez 2016 | oaxaco | randomised control | in preterm infants with very low birth weight (less than 1,500 g weight with gestational age of less than 34 weeks gestation |  | 24 | 20 | powder | 20 days | daily | Lactobacillus reuteri 5 drops, equivalent to 100 million colony forming units (1 × 108 CFU) . newborns with weight less than 1,000 g them 3 drops were administered Lactobacillus reuteri (60 million CFU) | Lactobacillus reuteri 5 drops, equivalent to 100 million colony forming units (1 × 108 CFU) . newborns with weight less than 1,000 g them 3 drops were administered Lactobacillus reuteri (60 million CFU) | no placebo |
| Hussain 2016 | Pakistan | Randomized control | Inclusion criteria: Pre-term neonates less than 36 weeks gestation, Low birth weight neonates< 2.5 Kg, Both genders, Both NG feed and bottle feed neonates, All neonates that were admitted at day 1 of life Exclusion: Neonates less than 30 weeks low birth weight neonates, less than 1.5 Kg ,Neonate on mechanical ventilatory support, IUGR (gestational age>36 weeks and weight<2.5kg), Patients with congenital cyanotic heart diseases or has birth asphyxia and persistent cyanosis and need of oxygen inhalation. | 45% | 150 | 150 | powder | 5 days | daily |  | Bifidobacteria | No probiotic supplementation |
| Kaban 2019 | Indonesia | double-blind randomized controlled clinical trial | quote: "This double-blind randomized controlled clinical trial compared the oral administration of L. reuteri DSM 17938 or placebo to neonates with a gestational age of 28–34 weeks and a birth weight of 1,000–1,800 g in a stable condition allowing the administration of oral and enteral nutrition and was conducted at the Neonatology Unit, Department of Pediatric Health of the Dr. Cipto Mangunkusumo Hospital from January to October 2017. The exclusion criteria were absolute contraindications for feeding such as lower gastrointestinal tract obstruction, massive gastrointestinal tract bleeding, NEC, sepsis and shock, and refusal of the infants' parents to participate in the study." | 52% | 47 | 47 | L. reuteri DSM 17938 (Interlac) suspension | at least 7 days or until the subject was discharged, experienced NEC, or died | five drops per day | 10^8 colony-forming units/day, the recommended dose | L. reuteri | placebo |
| Nandhini 2015 | India | prospective rct double blind | I: enterally fed, 28-34 weeks, bw>1000g; E: major congenital anomalies, surgical problems of the GI tract, severe birth asphyxia, early onset sepsis | NA | 110 | 110 | capsule into breastmilk | 7 days | twice daily | 1 capsule BID: Lactobacillus acidophilus 700 mil Cfu; bifidobacterium longum 400 mil CFU, lactobacillus rhamnosus 400 mil CFU, lactobacillus plantaris 300 mil CFU, lactobacillus casei 300 mil CFU, l. bulgaricus 300 mil CFU, B. breve 300 mil CFU and 100mg fructo-oligosaccharide | Lactobacillus acidophilus 700 mil Cfu; bifidobacterium longum 400 mil CFU, lactobacillus rhamnosus 400 mil CFU, lactobacillus plantaris 300 mil CFU, lactobacillus casei 300 mil CFU, l. bulgaricus 300 mil CFU, B. breve 300 mil CFU and 100mg fructo-oligosaccharide | breastmilk without supplementation |
| Niekerk 2015 | South africa | Randomized control | Included- birth weight of 500g and 1250g; (ii) were either HIV-exposed or HIV-unexposed; and (iii) received breast milk (either from their mothers or donor breast milk). Excluded if they had major abnormalities such as gastroschisis, a large omphalocele or congenital diaphragmatic hernia. | 62% in HIV exposed, 46% in HIV unexposed | 91 | 93 | powder | 4 weeks | daily | L. rhamnosus GG [0.35 x10^9 colony-forming units (CFU)] and B. infantis (0.35 x10^9 CFU) | Lactobacillus rhamnosus GG and Bifidobacterium infanti | Placebo |
| Oncel 2014 | Turkey | prospective rct double blind | I: <=32week, <=1500g birthweight, survived to feed enterally; e: major congenital malformations | 48.5 (194) | 200 | 200 | oil suspension | first feed to death or discharge | daily | 5 drops | Biogaia (l reuteri with 1x108 cfu total in five drops) | placebo same oil without biogaia |
| Panigrahi 2016 | India | community-based, double-blind, placebo-controlled randomized trial | Inclusion criteria included: neonate > 24 h and < 96 h old,  ≥ 2,000 g at birth, breastfeeding begun by 24 h of life, ability to tolerate oral feeds, informed consent by parent or guardian. Exclusion criteria were: evidence or suspicion of clinical sepsis before the infant was randomized, gestational age reported voluntarily by the mother to be < 35 weeks, infant > 96 h old, infant did not cry immediately after birth, mother had fever (> 38 °C) within 2 days of delivery, mother had foul-smelling amniotic discharge within 2 days of delivery, mother had abdominal tenderness within 2 days of delivery, amniotic fluid was meconium-stained, infant was on antibiotics, mother unlikely to stay in the village for 60 days, difficulty in carrying out study (maternal sickness etc.), or presence of major congenital anomalies (defined as any malformation that was felt to be life-threatening or that required surgical intervention). | 49.21% | 2,278 | 2,278 | Administered orally | 7 days | Daily |  | Lactobacillus plantarum | Placebo: 250 mg of maltodextrin |
| Rehman 2018 | Pakistan | randomised control | preterm infants having gestation of 27 to 36+7 weeks; they were VLBW (< 1500g) and they survived to feed enterally | 60.3 | 73 | 73 | powder |  | daily |  |  | placebo |
| Rojas 2012 | Colombia | multicenter, double-blinded, ran- domized, placebo-controlled trial | Preterm infants admitted to NICU, birth weight #2000 g, hemodynamically stable (blood pressure not requiring boluses or pressors), and #48 hours of age. Infants with evidence or suspicion of congenital intestinal obstruction or perforation, gastroschisis, large omphalocele, congenital diaphragmatic hernia, major congenital heart defects, or anticipated transfer to a NICU not participating in the study were excluded. | 50% | 372 | 378 | drops | until death or discharge from the NICU | daily | 5 drops | 108 colony-forming units of L reuteri DSM 17938 | placebo |
| Roy 2014 | India | prospective rct double blind | I: in NICU, stable oral feeding w/in 72 h birth, iconsent, <37weeks, <2500g, good renal and liver function, postnatal age <2week, no baseline fungal colonization from culture any site first 3 days of life, no antifungal px; E: major congenital malformation, risk factors for sepsis, signs of TORCH infection, cranial lesions on US, likely to die w/in 72 hours of birth, moms taking probiotics by capsule or powder | 73.2 (82) | 56 | 56 | expressed breast milk orally or OG | 6 weeks or until dischrged and feeding | daily | 6x 109 CFU lactobacillus: half a sachet of Lactobacillus acidophilus 1.25 billion, B. longum 0.125billion, B. bifidum 0.125billion, and B. lactis 1 billion / 1 gsachet | Prowel: Lactobacillus acidophilus 1.25 billion, B. longum 0.125billion, B. bifidum 0.125billion, and B. lactis 1 billion / 1 gsachet | sterile water in BM |
| Saengtawesin 2014 |  | Prospective, randomized control trial | All preterm infants with gestational age less than or equal to 34 weeks and birth weight less than or equal to 1,500 grams were randomized by blocks of four into two groups, study and control group. Very low birth weight preterm infants who had severe birth asphyxia, chromosome anomalies, cyanotic congenital heart disease, congenital intestinal obstruction, gastroschisis, omphalocele, nil per oral >3 weeks and parents who declined consent for study were excluded from the study. | 50.00% | 31 | 29 | Administered orally with breast milk or preterm formula | 6 weeks or until discharge | Twice daily | 125 mg/kg/dose | Infloran(1x10^9 Lactobacillus acidophilus and 1x109 Bifidobacterium bifida) | Placebo: unsupplemented breast milk or preterm formula |
| Samanta 2008 | India | prospective rct double blind | preterm (<32 weeks) VLBW (<1500g) born between Oct 2007-march 2008, started feed enterally, survived byond 48 hours of life; excluded "babies with major congenital and gi anomalies and babies who expired due to other neonatal illnesses" | NA | 91 | 95 | expressed breast milk | until discharge | twice daily | 2.5billion cfu each of 4 strains twice daily (125g/kg of milk or of bacteria?) | Bifidobacteria infantis, Bifidobacteria bifidum, Bifidobacteria longum, Lactobacillus acidophilus | breastmilk without supplementation |
| Sari 2011 | Turkey | randomised control | Inclusion Criteria: Infants with of gestational age of < 33 weeks or birth weight of o1500 g.  Exclusion Criteria: Major congenital malformationsand lack of parental consent | NA | 121 | 121 | Administered with Formual milk or breastmilk | Until discharge | daily | "Lactobacillus sporogenes with a dose of 350 000 000 c.f.u. once a day until discharge" | "Lactobacillus sporogenes | Standard of care |
| Serce 2013 | Turkey | randomised control | Inclusion Criteria: "VLBW infants (gestational age ≤32 weeks; birth weight ≤1500 g) who survived to feed enterally were eligible for the trial"  Exclusion Criteria: "Infants who had severe asphyxia (stage III), major congenital anomalies, those who had been fasted for more than 3 weeks, died in the first postnatal 14 days and infants who used antifungal therapy were excluded" | 46% | 104 | 104 | Intervention added to formula milk or breatmilk | Until discharge | Twice daily | "The study group received Saccharomyces boulardii (50 mg/kg equal to 0.5 × 109 cell/kg per dose | Saccharomyces boulardii | placebo (distilled water; 1 mL per dose twice daily) |
| Shadkam 2015 | Iran | randomised control | Inclusion Criteria: "premature infants admitted at\ the neonatal intensive care unit (NICU) during October 2012-March 2013. Gestational age of infants was estimated at 28-34 weeks using theDubowitz method, and birth weight of infants was calculated to be 1000-1800 grams."  -Exclusion Criteria:"presence of disorders such as digestive obstruction, GI bleeding, gastroschisis, omphalocele, withdrawal syndrome, neonatal proven or clinical sepsis, congenital heart defect and asphyxia (degree II or III)" | 50% | 30 | 30 |  | Until full enteral feedings | Twice daily | 5 ml of of 20 million live Lactobacillus reuteri protectis | g Lactobacillus reuteri DSM 17938 | "placebo group received 0.5 ml of distilled water every 12 hours". |
| Shashuhdar 2017 | India | randomised control | Inclusion Criteria: "All neonates with a birth weight between 750 g to 1499 g admitted to the NICU"  -Exclusion Criteria: "Neonates with gastrointestinal anomalies, severe congenital malformation, and those not started on enteral feeds by day 14 of life were excluded". | 54% | 25 | 32 | Liquid | Until discharge | Twice daily | dose of 1.25×109 CFU | Lactobacillus acidophilus, Lactobacillus rhamnosus, Bifidobacterium longum and Saccharomyces boulardii | "The no probiotic group received only breast milk and served as the control". |
| Sing 2017 | Nepal | randomised control | Inclusion Criteria: Preterm babies admitted to the NICU  -Exclusion Criteria: "Sick infants (neonates with clinical or proven sepsis), those with congenital malformation especially (central nervous system) malformation and other such as gastrointestinal obstruction, gastrointestinal bleeding,gastroschisis, omphalocoele, congenital heart defect and birth asphyxia (grade III). Out born babies were also excluded in this study". | 54 | 52 | 52 | Given with Breastmilk | Until reaching full enteral feeds | Twice daily | probiotics lactobacillus casei var. rhamnosis (LCR 35) 0.8 mg (half packet) dissolved in 2 ml of EBM in infant more than 1500 grams and 0.4 mg probiotics ( 1/4th packet) dissolved in 1 ml of EBM in infants less than 1500 grams | lactobacillus casei var. rhamnosi | "placebo as expressed breast milk only." |
| Sinha 2015 | India | randomised control | Inclusion Criteria: infants aged 3 days, born in the hospitals weighing 1500–2500 g"  -Exclusion Criteria: "extremely premature infants (<34 weeks), sick infants, those with congenital malformations incompatible with life, and those with guardians not giving consent and belonging to out of study areas" | 52% | 668 | 672 |  | 30 days | daily | dose of 10 billion cfu | (a mix of eight strains: Streptococcus thermophilus, Bifidobacterium breve, Bifidobacterium longum, Bifidobacterium infantis, Lactobacillus acidophilus, Lactobacillus plantarum, Lactobacillus paracasei and Lactobacillus delbrueckii spp bulgaricus |  |
| Tewari 2015 | India | randomised control | Inclusion Criteria:"Preterm neonates <34 weeks admitted to the NICU"  -Exclusion Criteria:  "i. Extramural preterm neonates >10 day age with clinical or lab marker of sepsis.ii. Preterm babies with necrotizing enterocolitis (NEC) or an intestinal surgical anomaly.iii. Preterm babies with a lethal congenital anomaly, dysmorphism or aneuploidy." | 49 | 123 | 121 | with the enteral feeds through orogastric tube | Postanatal age of 6 weeks | 2x109 spores in 5 ml minibottle in a dose of 2 ml per-oral every 8 hours mixed with the enteral feeds through orogastric tube | 2x109 spores in 5 ml minibottle in a dose of 2 ml per-oral every 8 hours | Bacillus clausii | "Babies in the placebo group received sterile water, 2 ml per-oral every 8 hours mixed with feeds" |
| Xu 2016 | China | randomised control | Inclusion Criteria: "hospital-born formula-fed infants with a gestational age of 30-37 weeks and a birth weight between 1500 and 2500 g".  -Exclusion Criteria:"severe neonatal pathologies, such as severe birth complications, GI malformations, chromosomal abnormalities, known immunodeficiency, hydrops fetalis, central venous catheter, antifungal drugs, and probiotics." | 50% | 63 | 62 | Adminsited separately from the formula | he study period ended at the 28th day after birth or when the infant was discharged from the hospital. Minimum duration of intervention was 7 days | Twice daily | 50 mg/kg. | Saccharomyces boulardii CNCM I-745 | " Nothing was administered to the control group" |

Supplementary table S 3: Subgroup analyses for probiotic supplementation during neonatal period

| Outcome or Subgroup | No. of Studies | Effect Estimate: relative Risk | Test for Subgroup Difference |
| --- | --- | --- | --- |
| All-cause mortality: Subgroup analysis: Settings | | | |
| Hospital based | 22 | 0.78 [0.65, 0.94] | p value=0.31 I^2^= 0 % |
| Community based | 3 | 1.25 [0.51, 3.05] |  |
| All-cause mortality: Subgroup analysis: Type of Probiotics | | | |
| Preparation contain a single strain of Probiotics | 9 | 0.80 [0.61, 1.05] | p value= 0.95 I^2^= 0 % |
| Preparation contained multiple strains of Probiotics | 12 | 0.80 [0.58, 1.09] |  |
| Preparation contained Synbiotics (Prebiotics + Probiotics) | 5 | 0.69 [0.29, 1.61] |  |
| All-cause mortality: Subgroup analysis: Type of participants | | | |
| Study include preterm/low birth weight babies | 24 | 0.79 [0.65, 0.95] | p value= 0.47 I^2^= 0 % |
| Study included term infants only | 1 | 1.38 [0.31, 6.08] |  |
| All-cause mortality: Subgroup analysis: Type of feedings | | | |
| Baby received breastmilk only | 14 | 0.81 [0.62, 1.05] | p value=0.44 I^2^= 0 % |
| Baby received formula milk only | 1 | 1.38 [0.31, 6.08] |  |
| Baby received both breastmilk and formula milk | 8 | 0.69 [0.48, 0.99] |  |
| Type of feeding was unclear | 3 | 1.33 [0.63, 2.81] |  |
| All-cause mortality: Subgroup analysis: Probiotics Preparation | | | |
| Preparation contained Lactobacillus | 10 | 0.82 [0.63, 1.05] | p value=0.47 I^2^= 0 % |
| Preparation contained Bifidobacterium | 1 | 0.43 [0.17, 1.09] |  |
| Preparation contained both Lactobacillus and Bifidobacterium | 13 | 0.71 [0.47, 1.08] |  |
| Preparation contained Saccharomyces boulardii only | 2 | 1.12 [0.46, 2.71] |  |
| Necrotizing Enterocolitis: Subgroup analysis: Probiotic preparation | | | |
| Preparation contained Lactobacillus | 13 | 0.39 [0.25, 0.61] | p value= 0.05 I^2^= 60.5 % |
| Preparation contained Bifidobacterium | 1 | 0.20 [0.09, 0.47] |  |
| Preparation contained both Lactobacillus and Bifidobacterium | 14 | 0.49 [0.36, 0.68] |  |
| Preparation contained Saccharomyces boulardii only | 2 | 0.94 [0.45, 1.95] |  |
| Necrotizing Enterocolitis: Subgroup analysis: Type of feeding | | | |
| Baby received breastmilk only | 13 | 0.43 [0.31, 0.59] | p value=0.74 I^2^= 0 % |
| Baby received formula only | 1 | 0.21 [0.03, 1.76] |  |
| Baby received both breastmilk and formula milk | 9 | 0.55 [0.33, 0.92] |  |
| Type of feeding was unclear | 7 | 0.41 [0.17, 1.00] |  |
| Necrotizing Enterocolitis: Subgroup analysis: Type of Probiotics | | | |
| Preparation contained a single strain of Probiotics | 12 | 0.48 [0.30, 0.76] | p value=0.50 I^2^= 0 % |
| Preparation contained multiple strains of Probiotics | 15 | 0.48 [0.35, 0.67] |  |
| Preparation contained Synbiotics (Prebiotics + Probiotics) | 3 | 0.28 [0.12, 0.67] |  |
| Neonatal Sepsis: Subgroup analysis: Probiotic preparation | | | |
| Preparation contained Lactobacillus | 11 | 0.74 [0.62, 0.87] | p value=0.79 I^2^= 0 % |
| Preparation contained Bifidobacterium | 1 | 0.81 [0.60, 1.09] |  |
| Preparation contained both Bifidobacterium and Lactobacillus | 6 | 0.83 [0.68, 1.02] |  |
| Preparation contained Saccharomyces boulardii only | 3 | 0.73 [0.57, 0.94] |  |
| Neonatal Sepsis: Subgroup analysis: Type of feeding | | | |
| Baby received breastmilk only | 8 | 0.71 [0.61, 0.83] | p value= 0.04 I^2^= 65 % |
| Baby received formula milk only | 2 | 0.59 [0.22, 1.56] |  |
| Baby received both formula and breastmilk only | 6 | 0.77 [0.65, 0.90] |  |
| Type of feeding was unclear | 4 | 0.95 [0.82, 1.09] |  |
| Neonatal Sepsis: Type of Probiotics | | | |
| Preparation contained single strain of Probiotics | 8 | 0.84 [0.74, 0.96] | p value= 0.21 I^2^= 35 % |
| Preparation contained multiple strains of Probiotics | 9 | 0.81 [0.68, 0.97] |  |
| Preparation contained Synbiotics (Prebiotics+Probiotics) | 4 | 0.67 [0.54, 0.83] |  |
| Neonatal Sepsis: Subgroup analysis: Settings | | | |
| Hospital Based | 19 | 0.83 [0.76, 0.91] | p value=0.19 I^2^= 42 % |
| Community based | 2 | 0.67 [0.49, 0.91] |  |

**Supplementary table S 4: Table of excluded studies**

| **Study** | **Reason for exclusion** |
| --- | --- |
| Abdulkadir 2016 [1] | Study conducted in a high income country (UK |
| Abrahamse-Berkeveld 2016 [2] | Study conducted in a high income country (Germany and Italy) |
| Abrahamsson 2005 [3] | No relevant outcomes were found |
| ADAPTS trial 2019 [4] | Wrong settings: Ongoing study in Australia |
| Agarwal 2003 [5] | No relevant clinical outcomes were reported |
| Agarwal 2017 [6] | Study conducted in a high income country (Australia) |
| Ahmadipour 2019[7] | Study conducted to treat neonatal Jaundice. |
| AhmadpourKacho 2005[8] | No relevant outcomes were reported |
| Al-Hosni 2012 [9] | Study conducted in a high income country (USA) |
| Ala-Houhala 1988 [10] | Study conducted in a high income country (Finland) |
| Allen 2010 [11] | Study conducted in high income country (UK) |
| Armanian 2014 [12] | Study participants were given only Prebiotics and no probiotics |
| Arthur 1992 [13] | Study population did not include Neonates |
| Aryayev 2018 [14] | Study conducted in high income country |
| Athalye-Jape 2018[15] | Study conducted in high income country |
| Awad 2010 [16] | Study was retracted |
| Ayah 2007 [17] | Vitamin A was given at 14 weeks |
| Aydin 2012 [18] | Population included children with Congential Heart disease only |
| Baglatzi 2016 [19] | Study conducted in a high income country |
| Bakker 2005 [20] | Study continued supplementation of probiotics for 4 months |
| Bakker Zierikzee 2005 [21] | Study conducted in a high income country (Netherland) |
| Bin-Nun 2005[22] | Study conducted in Israel |
| Bocquet 2013 [23] | Study conducted in a high income country (France) |
| Bora 2019 [24] | Study compared two forms of the same intervention |
| Cekola 2015 [25] | Study conducted in a high income country (USA) |
| Chabra 2013 [26] | Study conducted in a high income country (USA) |
| Chandel 2017 [27] | No relevant clinical outcomes were available |
| Chi 2019 [28] | No clinical outcomes were available |
| Chouraqui 2008[29] | Study conducted in a high income country (France) |
| Chrzanowska-Liszewska 2011[30] | Study conducted in a high income country (Poland) |
| Chua 2017[31] | Study conducted in a high income country (Netherland) |
| Corkins 2001[32] | Study conducted UK |
| Costalos 2003 [33] | Study conducted in a high income country (Greece) |
| Costeloe 2016 [34] | Study conducted in a high income country (UK) |
| Coutsoudis 1996 [35] | No clinical outcomes were available |
| Dani 2002 [36] | Study conducted in high income country |
| Darboe 2007 [37] | Study conducted on wrong study population (infants) |
| Delimont 2019 [38] | Study conducted on older children and used Sorghum-Based and Corn-Based Fortified Blended Foods |
| Delvin 2000 [39] | Study conducted in a high income country (US) |
| Deng 2010[40] | No abstract or full text available and no relevant outcomes were available. |
| Denkel 2017[41] | Study conducted in a high income country (Germany) |
| Deshpande 2016 [42] | Study conducted in high income country |
| Diaby 2018[43] | Observational study assessing the coverage of vitamin A supplementation |
| Dilli 2013[44] | Study conducted on wrong patient population (infants with congenital heart diseases) |
| Elom 2019 [45] | Wrong study population |
| Escribano 2018[46] | Study conducted in a high income country (Spain) |
| Galderisi 2016 [47] | The study investigated glucose monitoring and not the dextrose gel |
| Garg 2017 [48] | Wrong study design (retrospective cohort study) |
| Garland 2011 [49] | Study conducted in a high income country (Australia) |
| Garofoli 2014[50] | Wrong setting |
| Gomber 1996 [51] | Wrong study design |
| Gomez-Rodriguez 2019[52] | Compared two different regimens of probiotics. No placebo group was included. |
| Gonchar 2016[53] | No relevant outcomes were available. Study only available in the form of abstract. Authors were contacted for full text but no response |
| Guo-Qiang 2016[54] | Wrong study design |
| Hammerman 2007[55] | Wrong intervention |
| Harris 2016[56] | Study conducted in New Zealand |
| Hays 2015[57] | Study conducted in a high income country |
| Hoy-Schulz 2016[58] | Study conducted on wrong patient population |
| Hoyos 1999[59] | Wrong study design |
| Hua 2014[60] | Only abstract available and no relevant clinical outcomes were available |
| Huang 2016[61] | No relevant outcomes were available |
| Hunter 2012[62] | Wrong study design |
| Härtel 2019[63] | The study is being conducted in a high income country (Germany) |
| ICGPD 1996[64] | Study conducted in a high income country (Italy) |
| ICGPD 1996a[64] | Study conducted in a high income country (Italy) |
| Idindili 2007[65] | Study conducted on wrong patient population |
| Indrio 2008[66] | Study conducted in a high income country |
| IRCT 2015 [67] | Wrong comparison |
| Jacobs 2017 [68] | Study conducted in a high income country |
| Janvier 2014 [69] | Study conducted in a high income country |
| Kahbazi 2019[70] | Wrong study population |
| Kanic 2015[71] | Study conducted in a high income country |
| Karthikeyan 2017[72] | Study conducted in a high income country |
| Kiatchoosakun 2014[73] | No relevant outcomes were available |
| Kirkwood 2010[74] | Wrong study design |
| Kliegman 2005[75] | Wrong study design |
| Koksal 2015[76] | Only abstract was available and no analyzable data were reported |
| Kukkonen 2008[77] | Wrong study design |
| Leele 2015[78] | Study conducted in a high income country (Singapore) |
| Li 2019[79] | The intervention continued for 4 months |
| Lin 2009[80] | Study conducted in a high income country (Taiwan) |
| Long 2018[81] | Study conducted in a developed country |
| Lozano 2008[82] | Study conducted in a high income country |
| Lund 2014[83] | The control group did not receive the placebo but polio vaccine. It is difficult to tease out the effect of vitamin A supplementation vs. No vitamin A supplementation. |
| Lundelin 2017[84] | Study conducted in a high income country |
| Mactier 2012[85] | Study conducted in a high income country |
| Maldonado-Lobon 2015[86] | Study conducted in a high income country |
| Manzano 2017[87] | Study conducted in a high income country (Spain) |
| Manzoni 2006 [88] | Study conducted in a high income country (Italy) |
| Manzoni 2009 [89] | Study conducted in a high income country (italy) |
| Marissen 2019[90] | This is an ongoing study in Germany which is a high income country. |
| Martins 2009 [91] | Wrong study population |
| Materna 2010 [92] | Study conducted in a high income country |
| McCulloch 2012[93] | Study conducted in a high income country (UK) |
| McKinlay 2016[94] | Study conducted in a high income country |
| Meyer 2014 [95] | Study conducted in a high income country (Germany) |
| Mg 2011[96] | Study conducted in a high income country (Italy) |
| Mihatsch 2010[97] | Study conducted in a high income country (Germany) |
| Millar 2017[98] | Study conducted in a high income country |
| Moles 2015[99] | Study conducted in high income country |
| Nadella 2019[100] | Wrong intervention |
| Nct 2006[101] | Study conducted in high income country |
| Nct 2016 [102] | Wrong settings |
| Papagaroufalis 1991[103] | Study conducted in high income country (Greece) |
| Papagaroufalis 2014[104] | Study conducted in high income country (Greece) |
| Patole 2016[105] | Study conducted in a high income country (Australia) |
| Pearson 1992[106] | Study conducted in a high income country (USA) |
| Plummer 2018[107] | Study conducted in a high income country (Australia) |
| Puccio 2007[108] | Study conducted in a high income country (Italy) |
| Qiao 2017[109] | No relevant outcomes were available |
| Radke 2017[110] | Study conducted in a high income country (Germany) |
| Raguž 2016[111] | Wrong study design |
| Rakshasbhuvankar 2017[112] | Study conducted in a high income country (Australia) |
| Rawat 2016 [113] | Study conducted in the USA |
| Repa 2015[114] | Study conducted in a high income country (Austria) |
| Robbins 1993[115] | wrong comparator |
| Rodriguez-Herrera 2019[116] | Study conducted in a high income country |
| Rodríguez 2015[117] | Study compared two forms of Probiotics and no comparison with placebo was available |
| Rohan 2016[118] | Study conducted in a high income country (UK) |
| Rouge 2009[119] | Study conducted in a high income country (France) |
| Rubaltelli 2000[120] | Study conducted in a high income country (Italy) |
| Sadowska-Krawczenko 2012[121] | Study conducted in a high income country (Germany) |
| Samuels 2016 [122] | Study conducted in a high income country (Netherland) |
| Shenai 1987[123] | Study conducted in a high income country (USA) |
| Smilowitz 2017 [124] | Study conducted in a high income country (USA) |
| Storm 2019 [125] | Study conducted in a high income country USA |
| Stratiki 2007[126] | Study conducted in a high income country (Greece) |
| Strus 2018[127] | Study conducted in a high income country (Poland) |
| Ter 2017[128] | Study conducted in Australia |
| Thanhaeuser 2014 [129] | Study conducted in a high income country (Austria) |
| Totsu 2014[130] | Study conducted in a high income country (Japan) |
| Tyson 1999 [131] | Study conducted in a high income country (USA) |
| VAST study[132] | Study included children 6 months and older |
| Venkatarao 1996 [133] | Infant received vitamin A at 6 months |
| Vlieger 2009 [134] | Study conducted in high income country (Netherland) |
| Wardle 2001 [135] | Study conducted in high income country (UK) |
| West 1991[136] | Study included children 6-59 months of age |
| Yang 2011 [137] | Full text not available and no abstract was available either so no relevant outcomes were available |

**References:**

1. Abdulkadir, B.; Nelson, A.; Skeath, T.; Marrs, E.C.; Perry, J.D.; Cummings, S.P.; Embleton, N.D.; Berrington, J.E.; Stewart, C.J. Routine Use of Probiotics in Preterm Infants: Longitudinal Impact on the Microbiome and Metabolome. *Neonatology* **2016**, *109*, 239-247, doi:10.1159/000442936.

2. Abrahamse-Berkeveld, M.; Alles, M.; Franke-Beckmann, E.; Helm, K.; Knecht, R.; Kollges, R.; Sandner, B.; Knol, J.; Ben Amor, K.; Bufe, A. Infant formula containing galacto-and fructo-oligosaccharides and Bifidobacterium breve M-16V supports adequate growth and tolerance in healthy infants in a randomised, controlled, double-blind, prospective, multicentre study. *J Nutr Sci* **2016**, *5*, e42, doi:10.1017/jns.2016.35.

3. Abrahamsson, T.; Jakobsson, T.; Sinkiewicz, G.; Fredriksson, M.; Bjorksten, B. Intestinal microbiota in infants supplemented with the probiotic bacterium lactobacillus reuteri. *Journal of pediatric gastroenterology and nutrition 40(5):692* **2005**, *40*.

4. trial, A. ADAPTS trial. A Randomised Controlled Trial: Effect Of Probiotics On Gut Microbiome And Vaccine Responses In Newborns With Antibiotic-Induced Dysbiosis (ADAPTS: Antibiotic Dysbiosis and Probiotics Trial in infantS). ACTRN12619000369123p.

5. Agarwal, R.; Sharma, N.; Chaudhry, R.; Deorari, A.; Paul, V.K.; Gewolb, I.H.; Panigrahi, P. Effects of oral Lactobacillus GG on enteric microflora in low-birth-weight neonates. *J Pediatr Gastroenterol Nutr* **2003**, *36*, 397-402.

6. Agrawal, S.; Rao, S.; Nathan, L.; Patole, S. Effect of probiotics on C-reactive protein levels in preterm infants: Results of a randomised controlled trial. *Journal of Paediatrics and Child Health* **2017**, *53*, 3-4, doi:10.1111/jpc.13494_2.

7. Ahmadipour, S.; Baharvand, P.; Rahmani, P.; Hasanvand, A.; Mohsenzadeh, A. Effect of Synbiotic on the Treatment of Jaundice in Full Term Neonates: A Randomized Clinical Trial. *Pediatr Gastroenterol Hepatol Nutr* **2019**, *22*, 453-459, doi:10.5223/pghn.2019.22.5.453.

8. Ahmadpour, K.; Zahedpasha, Y.; Ahmadzadeh; Amiri, A.; Hajiahmadi, M.; Firoozi, M. Effect of vitamin A on prevention of retinopathy of prematurity (ROP). *Pediatric research* **2005**, *355*.

9. Al-Hosni, M.; Duenas, M.; Hawk, M.; Stewart, L.A.; Borghese, R.A.; Cahoon, M.; Atwood, L.; Howard, D.; Ferrelli, K.; Soll, R. Probiotics-supplemented feeding in extremely low-birth-weight infants. *Journal of perinatology : official journal of the California Perinatal Association* **2012**, *32*, 253-259, doi:10.1038/jp.2011.51.

10. Ala-Houhala M; Koskinen T; Mäki R; S., R. Serum vitamin A levels in mothers and their breast-fed term infants with or without supplemental vitamin A. *Acta Paediatr Scand* **1988 Mar**, *77*, 198-201.

11. Allen, S.J.; Martinez, E.G.; Gregorio, G.V.; Dans, L.F. Probiotics for treating acute infectious diarrhoea. *Cochrane Database Syst Rev* **2010**, 10.1002/14651858.CD003048.pub3, Cd003048, doi:10.1002/14651858.CD003048.pub3.

12. Armanian, A.M.; Sadeghnia, A.; Hoseinzadeh, M.; Mirlohi, M.; Feizi, A.; Salehimehr, N.; Torkan, M.; Shirani, Z. The effect of neutral oligosaccharides on fecal microbiota in premature infants fed exclusively with breast milk: A randomized clinical trial. *Journal of Research in Pharmacy Practice* **2016**, *5*, 27-34, doi:10.4103/2279-042X.176558.

13. Arthur P; Kirkwood B; Ross D; Morris S; Gyapong J; Tomkins A; H, A. Impact of vitamin A supplementation on childhood morbidity in northern Ghana. *Lancet (London, England)* **1992 Feb**, *8*, 361-362.

14. Aryayev, M.L.; Senkivska, L.I.; Bredeleva, N.K.; Talashova, I.V. Prophylaxis of acute respiratory infections via improving the immune system in late preterm newborns with E. coli strain Nissle 1917: a controlled pilot trial. *Pilot Feasibility Stud* **2018**, *4*, 79, doi:10.1186/s40814-018-0271-y.

15. Athalye-Jape, G.; Rao, S.; Patole, S. Effects of probiotics on experimental necrotizing enterocolitis: a systematic review and meta-analysis. *Pediatric research* **2018**, *83*, 16-22, doi:10.1038/pr.2017.218.

16. Awad, H.; Mokhtar, H.; Imam, S.S.; Gad, G.I.; Hafez, H.; Aboushady, N. Comparison between killed and living probiotic usage versus placebo for the prevention of necrotizing enterocolitis and sepsis in neonates. *Pakistan journal of biological sciences : PJBS* **2010**, *13*, 253-262.

17. Ayah RA; Mwaniki DL; Magnussen P; Tedstone AE; Marshall T; Alusala D; Luoba A; Kaestel P; Michaelsen KF; H, F. The effects of maternal and infant vitamin A supplementation on vitamin A status: a randomised trial in Kenya. *The British journal of nutrition* **2007 Aug**, *98*, 422-430.

18. Aydin, B.; Dilli, D.; Erol, S.; Ozyazici, E.; Beken, S.; Cullas Ilarslan, N.E.; Zenciroglu, A.; Okumus, N. The effects of synbiotics on morbidity and mortality in newborns with cyanotic congenital heart disease: a prospective randomized controlled trial. *Archives of disease in childhood* **2012**, *97*, A462‐, doi:10.1136/archdischild-2012-302724.1633.

19. Baglatzi, L.; Gavrili, S.; Stamouli, K.; Zachaki, S.; Favre, L.; Pecquet, S.; Benyacoub, J.; Costalos, C. Effect of Infant Formula Containing a Low Dose of the Probiotic Bifidobacterium lactis CNCM I-3446 on Immune and Gut Functions in C-Section Delivered Babies: A Pilot Study. *Clinical medicine insights. Pediatrics* **2016**, *10*, 11-19, doi:10.4137/CMPed.S33096.

20. Bakker-Zierikzee, A.M.; Alles, M.S.; Knol, J.; Kok, F.; Tolboom, J.J.M.; Bindels, J.G. Prebiotic oligosaccharides in infant nutrition: addition of only galactooligosaccharides does not induce faecal acidic pH and SCFA-spectrum typical for breast fed infants. *Journal of pediatric gastroenterology and nutrition* **2005**, *40*, 693.

21. Bakker-Zierikzee, A.M.; Alles, M.S.; Knol, J.; Kok, F.J.; Tolboom, J.J.; Bindels, J.G. Effects of infant formula containing a mixture of galacto- and fructo-oligosaccharides or viable Bifidobacterium animalis on the intestinal microflora during the first 4 months of life. *The British journal of nutrition* **2005**, *94*, 783-790.

22. Bin-Nun, A.; Bromiker, R.; Wilschanski, M.; Kaplan, M.; Rudensky, B.; Caplan, M.; Hammerman, C. Oral probiotics prevent necrotizing enterocolitis in very low birth weight neonates. *The Journal of pediatrics* **2005**, *147*, 192-196, doi:10.1016/j.jpeds.2005.03.054.

23. Bocquet, A.; Lachambre, E.; Kempf, C.; Beck, L. Effect of infant and follow-on formulas containing B lactis and galacto- and fructo-oligosaccharides on infection in healthy term infants. *J Pediatr Gastroenterol Nutr* **2013**, *57*, 180-187, doi:10.1097/MPG.0b013e318297f35e.

24. Bora, R.; Deori, S. Transitional Hypoglycaemia Management in Small for Gestational Age Neonates with Sucrose Enriched Expressed Breastmilk in Resource Poor Setting. *J Trop Pediatr* **2019**.

25. Cekola, P.L.; Czerkies, L.A.; Storm, H.M.; Wang, M.H.; Roberts, J.; Saavedra, J.M. Growth and Tolerance of Term Infants Fed Formula With Probiotic Lactobacillus reuteri. *Clinical pediatrics* **2015**, *54*, 1175-1184, doi:10.1177/0009922815574076.

26. Chabra, S.; Mayock, D.E.; Zerzan, J.; Bittner, R.; Neufeld, M.D.; Gleason, C.A. Vitamin A status after prophylactic intramuscular vitamin A supplementation in extremely low birth weight infants. *Nutrition in clinical practice : official publication of the American Society for Parenteral and Enteral Nutrition* **2013**, *28*, 381-386, doi:10.1177/0884533613479132.

27. Chandel, D.S.; Perez-Munoz, M.E.; Yu, F.; Boissy, R.; Satpathy, R.; Misra, P.R.; Sharma, N.; Chaudhry, R.; Parida, S.; Peterson, D.A., et al. Changes in the Gut Microbiota After Early Administration of Oral Synbiotics to Young Infants in India. *J Pediatr Gastroenterol Nutr* **2017**, *65*, 218-224, doi:10.1097/mpg.0000000000001522.

28. Chi, C.; Xue, Y.; Liu, R.; Wang, Y.; Lv, N.; Zeng, H.; Buys, N.; Zhu, B.; Sun, J.; Yin, C. Effects of a formula with a probiotic Bifidobacterium lactis Supplement on the gut microbiota of low birth weight infants. *European journal of nutrition* **2019**, 10.1007/s00394-019-02006-4, doi:10.1007/s00394-019-02006-4.

29. Chouraqui, J.P.; Grathwohl, D.; Labaune, J.M.; Hascoet, J.M.; de Montgolfier, I.; Leclaire, M.; Giarre, M.; Steenhout, P. Assessment of the safety, tolerance, and protective effect against diarrhea of infant formulas containing mixtures of probiotics or probiotics and prebiotics in a randomized controlled trial. *The American journal of clinical nutrition* **2008**, *87*, 1365-1373, doi:10.1093/ajcn/87.5.1365.

30. Chrzanowska-Liszewska D; Seliga-Siwecka J; l., K.M. The effect of Lactobacillus rhamnosus GG supplemented enteral feeding on the microbiotic flora of preterm infants-double blinded randomized control trial. *Early human development* **2012 Jan**, *88*, 57-60.

31. Chua, M.C.; Ben-Amor, K.; Lay, C.; Neo, A.G.E.; Chiang, W.C.; Rao, R.; Chew, C.; Chaithongwongwatthana, S.; Khemapech, N.; Knol, J., et al. Effect of Synbiotic on the Gut Microbiota of Cesarean Delivered Infants: A Randomized, Double-blind, Multicenter Study. *J Pediatr Gastroenterol Nutr* **2017**, *65*, 102-106, doi:10.1097/mpg.0000000000001623.

32. Corkins, M.R. Randomised controlled trial of oral vitamin A supplementation in preterm infants to prevent chronic lung disease. *Nutrition in Clinical Practice* **2001**, *16*, 265-265.

33. Costalos, C.; Skouteri, V.; Gounaris, A.; Sevastiadou, S.; Triandafilidou, A.; Ekonomidou, C.; Kontaxaki, F.; Petrochilou, V. Enteral feeding of premature infants with Saccharomyces boulardii. *Early human development* **2003**, *74*, 89-96.

34. Costeloe, K.; Hardy, P.; Juszczak, E.; Wilks, M.; Millar, M.R. Bifidobacterium breve BBG-001 in very preterm infants: a randomised controlled phase 3 trial. *Lancet (London, England)* **2016**, *387*, 649-660, doi:10.1016/s0140-6736(15)01027-2.

35. Coutsoudis, A.; Adhikari, M.; Pillay, K.; Coovadia, H.M. Absorption of high-dose enteral vitamin A in low-birth-weight neonates. *South African medical journal = Suid-Afrikaanse tydskrif vir geneeskunde* **1996**, *86*, 1337-1339.

36. Dani, C.; Biadaioli, R.; Bertini, G.; Martelli, E.; Rubaltelli, F.F. Probiotics feeding in prevention of urinary tract infection, bacterial sepsis and necrotizing enterocolitis in preterm infants. A prospective double-blind study. *Biology of the neonate* **2002**, *82*, 103-108, doi:10.1159/000063096.

37. Darboe, M.K.; Thurnham, D.I.; Morgan, G.; Adegbola, R.A.; Secka, O.; Solon, J.A.; Jackson, S.J.; Northrop-Clewes, C.; Fulford, T.J.; Doherty, C.P., et al. Effectiveness of an early supplementation scheme of high-dose vitamin A versus standard WHO protocol in Gambian mothers and infants: a randomised controlled trial. *Lancet (London, England)* **2007**, *369*, 2088-2096, doi:10.1016/s0140-6736(07)60981-7.

38. Delimont, N.M.; Vahl, C.I.; Kayanda, R.; Msuya, W.; Mulford, M.; Alberghine, P.; Praygod, G.; Mngara, J.; Alavi, S.; L, L.B. Complementary Feeding of Sorghum-Based and Corn-Based Fortified Blended Foods Results in Similar Iron, Vitamin A, and Anthropometric Outcomes in the MFFAPP Tanzania Efficacy Study. *Current Developments in Nutrition* **2019**, *3*.

39. Delvin, E.E.; Salle, B.L.; Claris, O.; Putet, G.; Hascoet, J.M.; Desnoulez, L.; Messai, S.; Levy, E. Oral vitamin A, E and D supplementation of pre-term newborns either breast-fed or formula-fed: a 3-month longitudinal study. *J Pediatr Gastroenterol Nutr* **2005**, *40*, 43-47.

40. J, D.; K, C. Early minimal feeding combined with probiotics toprevent necrotizing enterocolitis in preterm infant. *Chinese Journal of Modern Drug Application* **2010**, *4*, 13-14.

41. Denkel, L.A.; Schwab, F.; Garten, L.; Geffers, C.; Gastmeier, P.; Piening, B. Dual-strain probiotics reduce NEC, mortality and neonatal bloodstream infections among extremely low birthweight infants. *Archives of disease in childhood. Fetal and neonatal edition* **2017**, *102*, F559-f560, doi:10.1136/archdischild-2017-313897.

42. Deshpande, G.; Rao, S.; Athalye-Jape, G.; Conway, P.; Patole, S. Probiotics in very preterm infants: the PiPS trial. *Lancet (London, England)* **2016**, *388*, 655, doi:10.1016/s0140-6736(16)31271-5.

43. Diaby, A.; Mohamed, A.S.; Camara, B.; Sall, G.; Youssouf, M. Coverage of vitamin A supplementation in children 6 to 59 months after two successive campaigns in Senegal. *Journal de Pediatrie et de Puericulture* **2018**, *6*, 277-281.

44. Dilli, D.; Aydin, B.; Zenciroglu, A.; Ozyazici, E.; Beken, S.; Okumus, N. Treatment outcomes of infants with cyanotic congenital heart disease treated with synbiotics. *Pediatrics* **2013**, *132*, e932-938, doi:10.1542/peds.2013-1262.

45. Elom, M.O.; Eyo, J.E.; Okafor, F.C.; Nworie, A.; Usanga, V.U.; Attamah, G.N.; Igwe, C.C. Improved infant hemoglobin (Hb) and blood glucose concentrations: The beneficial effect of maternal vitamin A supplementation of malaria-infected mothers in Ebonyi State, Nigeria. *Pathogens and global health* **2017**, *111*, 45-48, doi:10.1080/20477724.2016.1261489.

46. Escribano, J.; Ferre, N.; Gispert-Llaurado, M.; Luque, V.; Rubio-Torrents, C.; Zaragoza-Jordana, M.; Polanco, I.; Codoner, F.M.; Chenoll, E.; Morera, M., et al. Bifidobacterium longum subsp infantis CECT7210-supplemented formula reduces diarrhea in healthy infants: a randomized controlled trial. *Pediatric research* **2018**, *83*, 1120-1128, doi:10.1038/pr.2018.34.

47. Galderisi, A.; Facchinetti, A.; Steil, G.M.; Ortiz-Rubio, P.; Cobelli, C.; Trevisanuto, D. Neonatal hypoglycemia continuous glucose monitoring: A randomized controlled trial in preterm infants. *Diabetes Technology and Therapeutics* **2016**, A57.

48. Garg, B.D.; Kabra, N.S.; Balasubramanian, H.; Avasthi, B.S.; Sharma, S.R.; Ahmed, J.; Dash, S.K. Prophylactic probiotics for prevention of necrotizing enterocolitis in preterm neonates: A cohort study. *Perinatology* **2017**, *18*, 41-49.

49. Garland, S.M.; Tobin, J.M.; Pirotta, M.; Tabrizi, S.N.; Opie, G.; Donath, S.; Tang, M.L.; Morley, C.J.; Hickey, L.; Ung, L., et al. The ProPrems trial: investigating the effects of probiotics on late onset sepsis in very preterm infants. *BMC Infect Dis* **2011**, *11*, 210, doi:10.1186/1471-2334-11-210.

50. Garofoli, F.; Civardi, E.; Indrio, F.; Mazzucchelli, I.; Angelini, M.; Tinelli, C.; Stronati, M. The early administration of Lactobacillus reuteri DSM 17938 controls regurgitation episodes in full-term breastfed infants. *International journal of food sciences and nutrition* **2014**, *65*, 646-648, doi:10.3109/09637486.2014.898251.

51. Gomber, S. Potential toxicity of vitamin A supplementation in infancy. *Indian pediatrics* **1996**, *33*, 1065-1067.

52. Gomez-Rodriguez, G.; Amador-Licona, N.; Daza-Benitez, L.; Barbosa-Sabanero, G.; Carballo-Magdaleno, D.; Aguilar-Padilla, R.; Gonzalez-Ramirez, E. Single strain versus multispecies probiotic on necrotizing enterocolitis and faecal IgA levels in very low birth weight preterm neonates: A randomized clinical trial. *Pediatrics and neonatology* **2019**, *60*, 564-569, doi:10.1016/j.pedneo.2019.02.005.

53. Gonchar, N.V.; Lo Schiavo, L.A.; Suvorov, A.N.; Grigoriev, S.G. Forecast successful prevention of infectious complications in preterm infants. *Jurnal Infektologii* **2016**, *8*, 32-37.

54. Guo-Qiang, Z.; Hua-Jian, H.; Chuan-Yang, L.; Shristi, S.; Zhong-Yue, L. Probiotics for Preventing Late-Onset Sepsis in Preterm Neonates. *Medicine* **2016**, *95*, 1-11, doi:10.1097/md.0000000000002581.

55. Hammerman, C.; Bin-nun, A. Prebiotics vs. Placebo in the Prevention of Necrotizing Enterocolitis in Premature Neonates. *Clinicaltrials.gov identifier: NCT00437567* **2007**.

56. Harris, R.J. Is milk best for infants and toddlers? *Journal of Nutritional & Environmental Medicine* **2002**, *12*, 145-151.

57. Hays, S.; Jacquot, A.; Gauthier, H.; Kempf, C.; Beissel, A.; Pidoux, O.; Jumas-Bilak, E.; Decullier, E.; Lachambre, E.; Beck, L., et al. Probiotics and growth in preterm infants: a randomized controlled trial, PREMAPRO study. *Clinical nutrition (Edinburgh, Scotland)* **2015**, 10.1016/j.clnu.2015.06.006, doi:10.1016/j.clnu.2015.06.006.

58. Hoy-Schulz, Y.E.; Jannat, K.; Roberts, T.; Zaidi, S.; Rahman, M.; Unicomb, L.; Luby, S.; Parsonnet, J. Colonization of probiotics in Bangladeshi infants after different administration regimens. *Open forum infectious diseases* **2016**, *3*, doi:10.1093/ofid/ofw172.1056.

59. Hoyos, A.B. Reduced incidence of necrotizing enterocolitis associated with enteral administration of Lactobacillus acidophilus and Bifidobacterium infantis to neonates in an intensive care unit. *International journal of infectious diseases : IJID : official publication of the International Society for Infectious Diseases* **1999**, *3*, 197-202.

60. Hua, X.T.; Tang, J.; Mu, D.Z. Effect of oral administration of probiotics on intestinal colonization with drug-resistant bacteria in preterm infants. *Chinese journal of contemporary pediatrics* **2014**, *16*, 606‐609, doi:10.7499/j.issn.1008-8830.2014.06.009.

61. Huang, N.N.; Wang, G.Z.; Wang, J.F.; Yuan, Y.X. Risk factors for neonatal nosocomial enteric infection and the effect of intervention with BIFICO. *Eur Rev Med Pharmacol Sci* **2016**, *20*, 3713-3719.

62. Hunter, C.; Dimaguila, M.A.; Gal, P.; Wimmer, J.E., Jr.; Ransom, J.L.; Carlos, R.Q.; Smith, M.; Davanzo, C.C. Effect of routine probiotic, Lactobacillus reuteri DSM 17938, use on rates of necrotizing enterocolitis in neonates with birthweight < 1000 grams: a sequential analysis. *BMC pediatrics* **2012**, *12*, 142, doi:10.1186/1471-2431-12-142.

63. Härtel., C. PRIMAL Clinical Study: Efficacy of probiotics to prevent gut dysbiosis in very preterm infants (VPIs) and moderate preterm infants of 28+0 – 32+6 weeks of gestation: A randomized, placebo-controlled double-blind study. [*http://www.drks.de/DRKS00013197DRKS00013197*](http://www.drks.de/DRKS00013197DRKS00013197)*.*

64. Vitamin A supplementation in premature neonates with postnatal lung injury. Italian Collaborative Group on Preterm Delivery (ICGPD). *International journal of clinical pharmacology and therapeutics* **1996**, *34*, 362-365.

65. Idindili, B.; Masanja, H.; Urassa, H.; Bunini, W.; van Jaarsveld, P.; Aponte, J.J.; Kahigwa, E.; Mshinda, H.; Ross, D.; Schellenberg, D.M. Randomized controlled safety and efficacy trial of 2 vitamin A supplementation schedules in Tanzanian infants. *The American journal of clinical nutrition* **2007**, *85*, 1312-1319, doi:10.1093/ajcn/85.5.1312.

66. Indrio, F.; Riezzo, G.; Raimondi, F.; Bisceglia, M.; Cavallo, L.; Francavilla, R. The effects of probiotics on feeding tolerance, bowel habits, and gastrointestinal motility in preterm newborns. *The Journal of pediatrics* **2008**, *152*, 801-806, doi:10.1016/j.jpeds.2007.11.005.

67. Irct201505253915N. comparing the efficacy of two doses of vitamin A in preterm infants. <http://www.who.int/trialsearch/Trial2.aspx>? 2015. [Other: IRCT201505253915N].

68. Jacobs, S.E.; Hickey, L.; Donath, S.; Opie, G.F.; Anderson, P.J.; Garland, S.M.; Cheong, J.L.Y. Probiotics, prematurity and neurodevelopment: follow-up of a randomised trial. *BMJ Paediatr Open* **2017**, *1*, e000176, doi:10.1136/bmjpo-2017-000176.

69. Janvier, A.; Malo, J.; Barrington, K.J. Cohort study of probiotics in a North American neonatal intensive care unit. *Obstetrical and Gynecological Survey* **2014**, *69*, 460-461, doi:10.1097/01.ogx.0000453820.46858.73.

70. Kahbazi, M., Sharafkhah, M., Yousefichaijan, P., Taherahmadi, H., Rafiei, M., Kaviani, P., Abaszadeh, S., Massoudifar, A., Mohammadbeigi, A. Vitamin A supplementation is effective for improving the clinical symptoms of urinary tract infections and reducing renal scarring in girls with acute pyelonephritis: a randomized, double-blind placebo-controlled, clinical trial study. *Complementary therapies in medicine* **2019**, *42*, 429-437.

71. Kanic, Z.; Micetic Turk, D.; Burja, S.; Kanic, V.; Dinevski, D. Influence of a combination of probiotics on bacterial infections in very low birthweight newborns. *Wiener klinische Wochenschrift* **2015**, *127 Suppl 5*, S210-215, doi:10.1007/s00508-015-0845-0.

72. Karthikeyan, G.; Bhat, B.V. The PiPS (Probiotics in Preterm Infants Study) Trial - Controlling the Confounding Factor of Cross-contamination Unveils Significant Benefits. *Indian pediatrics* **2017**, *54*, 162.

73. Kiatchoosakun, P.; Jirapradittha, J.; Panthongviriyakul, C.; Khampitak, T.; Yongvanit, P.; Boonsiri, P. Vitamin a supplementation for prevention of bronchopulmonary dysplasia in very-low-birth-weight premature thai infants: a randomized trial. *Chotmaihet thangphaet [journal of the medical association of thailand]* **2014**, *97*, S82‐S88.

74. Kirkwood, B.; Humphrey, J.; Moulton, L.; Martines, J. Neonatal vitamin A supplementation and infant survival. *Lancet (London, England)* **2010**, *376*, 1643-1644, doi:10.1016/s0140-6736(10)61895-8.

75. Kliegman, R.M.; Willoughby, R.E. Prevention of necrotizing enterocolitis with probiotics. *Pediatrics* **2005**, *115*, 171-172, doi:10.1542/peds.2004-2271.

76. Koksal, N.; Varal, I.; Ozkan, H.; Bagci, O.; Dotan, P. Effect of probiotic support on feeding intolerance and mortality at preterm infants. *Journal of perinatal medicine* **2015**, *43*, doi:10.1515/jpm-2015-2003.

77. Kukkonen, K.; Savilahti, E.; Haahtela, T.; Juntunen-Backman, K.; Korpela, R.; Poussa, T.; Tuure, T.; Kuitunen, M. Long-term safety and impact on infection rates of postnatal probiotic and prebiotic (synbiotic) treatment: randomized, double-blind, placebo-controlled trial. *Pediatrics* **2008**, *122*, 8-12, doi:10.1542/peds.2007-1192.

78. Lee le, Y.; Bharani, R.; Biswas, A.; Lee, J.; Tran, L.A.; Pecquet, S.; Steenhout, P. Normal growth of infants receiving an infant formula containing Lactobacillus reuteri, galacto-oligosaccharides, and fructo-oligosaccharide: a randomized controlled trial. *Maternal health, neonatology and perinatology* **2015**, *1*, 9, doi:10.1186/s40748-015-0008-3.

79. Li, L.; Han, Z.; Niu, X.; Zhang, G.; Jia, Y.; Zhang, S.; He, C. Probiotic Supplementation for Prevention of Atopic Dermatitis in Infants and Children: A Systematic Review and Meta-analysis. *American Journal of Clinical Dermatology* **2019**, *20*, 367-377, doi:10.1007/s40257-018-0404-3.

80. Lin, W.H.; Lin, C.K.; Sheu, S.J.; Hwang, C.F.; Ye, W.T.; Hwang, W.Z.; Tsen, H.Y. Antagonistic activity of spent culture supernatants of lactic acid bacteria against Helicobacter pylori growth and infection in human gastric epithelial AGS cells. *Journal of food science* **2009**, *74*, M225-230, doi:10.1111/j.1750-3841.2009.01194.x.

81. Long, A.; Dempsey, E. Oral probiotic supplementation in the prevention of necrotising enterocolitis among very preterm infants. *Irish Journal of Medical Science* **2018**, *187*, S75, doi:10.1007/s11845-018-1833-y.

82. Lozano, J.M. Prophylactic Probiotics for the Prevention of Sepsis and NEC in Premature Infants in Colombia. A Randomized Double-Blind, Multicenter Trial. *Clinicaltrials.gov identifier: NCT00727363.* **2008**.

83. Lund, N.; Biering-Sorensen, S.; Andersen, A.; Monteiro, I.; Camala, L.; Jorgensen, M.J.; Aaby, P.; Benn, C.S. Neonatal vitamin A supplementation associated with a cluster of deaths and poor early growth in a randomised trial among low-birth-weight boys of vitamin A versus oral polio vaccine at birth. *BMC pediatrics* **2014**, *14*, 214, doi:10.1186/1471-2431-14-214.

84. Lundelin, K.; Salminen, S.; Isolauri, E. Long-term safety and efficacy of perinatal probiotic intervention: Evidence from a follow up study. *World Allergy Organization Journal* **2017**, *10*, doi:10.1186/s40413-017-0170-3.

85. Mactier, H.; McCulloch, D.L.; Hamilton, R.; Galloway, P.; Bradnam, M.S.; Young, D.; Lavy, T.; Farrell, L.; Weaver, L.T. Vitamin A supplementation improves retinal function in infants at risk of retinopathy of prematurity. *The Journal of pediatrics* **2012**, *160*, 954-959.e951, doi:10.1016/j.jpeds.2011.12.013.

86. Maldonado-Lobon, J.A.; Diaz-Lopez, M.A.; Carputo, R.; Duarte, P.; Diaz-Ropero, M.P.; Valero, A.D.; Sanudo, A.; Sempere, L.; Ruiz-Lopez, M.D.; Banuelos, O., et al. Lactobacillus fermentum CECT 5716 Reduces Staphylococcus Load in the Breastmilk of Lactating Mothers Suffering Breast Pain: A Randomized Controlled Trial. *Breastfeeding medicine : the official journal of the Academy of Breastfeeding Medicine* **2015**, *10*, 425-432, doi:10.1089/bfm.2015.0070.

87. Manzano, S.; De Andres, J.; Castro, I.; Rodriguez, J.M.; Jimenez, E.; Espinosa-Martos. Safety and tolerance of three probiotic strains in healthy infants: a multi-centre randomized, double-blind, placebo-controlled trial. *Benef Microbes* **2017**, *8*, 569-578.

88. Manzoni, P.; Mostert, M.; Leonessa, M.L.; Priolo, C.; Farina, D.; Monetti, C.; Latino, M.A.; Gomirato, G. Oral supplementation with Lactobacillus casei subspecies rhamnosus prevents enteric colonization by Candida species in preterm neonates: a randomized study. *Clinical infectious diseases : an official publication of the Infectious Diseases Society of America* **2006**, *42*, 1735-1742, doi:10.1086/504324.

89. Manzoni, P.; Rinaldi, M.; Cattani, S.; Pugni, L.; Romeo, M.G.; Messner, H.; Stolfi, I.; Decembrino, L.; Laforgia, N.; Vagnarelli, F., et al. Bovine lactoferrin supplementation for prevention of late-onset sepsis in very low-birth-weight neonates: a randomized trial. *Jama* **2009**, *302*, 1421-1428, doi:10.1001/jama.2009.1403.

90. Marissen, J.; Haiss, A.; Meyer, C.; Van Rossum, T.; Bunte, L.M.; Frommhold, D.; Gille, C.; Goedicke-Fritz, S.; Gopel, W.; Hudalla, H., et al. Efficacy of Bifidobacterium longum, B. infantis and Lactobacillus acidophilus probiotics to prevent gut dysbiosis in preterm infants of 28+0-32+6 weeks of gestation: a randomised, placebo-controlled, double-blind, multicentre trial: the PRIMAL Clinical Study protocol. *BMJ open* **2019**, *9*, e032617, doi:10.1136/bmjopen-2019-032617.

91. Martins, C.L.; Benn, C.S.; Andersen, A.; Bale, C.; Schaltz-Buchholzer, F.; Do, V.A.; Rodrigues, A.; Aaby, P.; Ravn, H.; Whittle, H., et al. A randomized trial of a standard dose of Edmonston-Zagreb measles vaccine given at 4.5 months of age: effect on total hospital admissions. *The Journal of infectious diseases* **2014**, *209*, 1731-1738, doi:10.1093/infdis/jit804.

92. Materna, L. Evaluation of the Effect of Milk Based Infant Formula Supplemented Either With Probiotic Microorganisms and/or With Prebiotic on the Intestinal Microflora During the First 4 Months of Life of Healthy, Full Term Infants and it's Long Term Effect on Morbidity up to the Age of 9 Months. *Clinicaltrials.gov identifier: NCT00836771* **2010**.

93. McCulloch, D.L.; Mactier, H.; Farrell, L.; Hamilton, R. Light-adapted ERGs in the VitAL study: a randomised controlled trial of early high-dose vitamin A in infants at risk of retinopathy of prematurity (ROP). *Documenta ophthalmologica.* **2012**, *124*, 22‐23, doi:10.1007/s10633-012-9332-3.

94. McKinlay, C.J.; Rebello, C.; Tarnow-Mordi, W. Probiotics in very preterm infants: the PiPS trial. *Lancet (London, England)* **2016**, *388*, 655, doi:10.1016/s0140-6736(16)31270-3.

95. Meyer, M.P.; Alexander, T. Use of the probiotic lactobacillus GG in combination with lactoferrin and improved outcomes in preterm infants. *Journal of Paediatrics and Child Health* **2015**, *51*, 69, doi:10.1111/jpc.12884-4.

96. Mg, R.; Dm, R.; L, T.; S, O.; F, P.; F, C. Role of probiotics in the prevention of the enteric colonization by Candida in preterm newborns: incidence of late-onset sepsis and neurological outcome. *Journal of perinatology* **2011**, *31*, 63–69.

97. Mihatsch, W.; Pohlandt, F. [Should probiotics already be recommended as standard clinical care for preterm infants with birth weights below 1,500 g?]. *Klin Padiatr* **2010**, *222*, 282-283, doi:10.1055/s-0030-1263151.

98. Millar, M.; Seale, J.; Greenland, M.; Hardy, P.; Juszczak, E.; Wilks, M.; Panton, N.; Costeloe, K.; Wade, W.G. The Microbiome of Infants Recruited to a Randomised Placebo-controlled Probiotic Trial (PiPS Trial). *EBioMedicine* **2017**, *20*, 255-262, doi:10.1016/j.ebiom.2017.05.019.

99. Moles, L.; Escribano, E.; de Andres, J.; Montes, M.T.; Rodriguez, J.M.; Jimenez, E.; Saenz de Pipaon, M.; Espinosa-Martos, I. Administration of Bifidobacterium breve PS12929 and Lactobacillus salivarius PS12934, two strains isolated from human milk, to very low and extremely low birth weight preterm infants: a pilot study. *Journal of immunology research* **2015**, *2015*, 538171, doi:10.1155/2015/538171.

100. Nadella, P.; Smith, E.R.; Muhihi, A.; Noor, R.A.; Masanja, H.; Fawzi, W.W.; Sudfeld, C.R. Determinants of delayed or incomplete diphtheria-tetanus-pertussis vaccination in parallel urban and rural birth cohorts of 30,956 infants in Tanzania. *BMC Infectious Diseases* **2019**, *19*, 188.

101. Nct. Influence of Probiotics on Prevention of Atopy, Atopic Disease and Immunological Responses. [*Https://clinicaltrials.gov/show/nct00318695*](Https://clinicaltrials.gov/show/nct00318695) **2006**.

102. Nct. Functional Evaluation of Two Infant Formula Supplemented With Probiotics Isolated From Breast Milk. [*Https://clinicaltrials.gov/show/nct03204630*](Https://clinicaltrials.gov/show/nct03204630) **2016**.

103. Papagaroufalis, C.; Megreli, C.; Hagjigeorgi, C.; Xanthou, M. A trial of vitamin A supplementation for the prevention of intraventricular hemorrhage in very low birth weight neonates. *Journal of perinatal medicine* **1991**, *19 Suppl 1*, 382-387.

104. Papagaroufalis, K.; Fotiou, A.; Egli, D.; Tran, L.A.; Steenhout, P. A Randomized Double Blind Controlled Safety Trial Evaluating d-Lactic Acid Production in Healthy Infants Fed a Lactobacillus reuteri-containing Formula. *Nutrition and metabolic insights* **2014**, *7*, 19-27, doi:10.4137/nmi.S14113.

105. Patole, S.K.; Keil, A.D.; Nathan, E.; Doherty, D.; Esvaran, M.; Simmer, K.N.; Conway, P. Effect of Bifidobacterium breve M-16V supplementation on faecal bifidobacteria in growth restricted very preterm infants - analysis from a randomised trial. *The journal of maternal-fetal & neonatal medicine : the official journal of the European Association of Perinatal Medicine, the Federation of Asia and Oceania Perinatal Societies, the International Society of Perinatal Obstet* **2016**, *29*, 3751-3755, doi:10.3109/14767058.2016.1147554.

106. Pearson, E.; Bose, C.; Snidow, T.; Stiles, A.; Ransom, L.; Young, T. A trial of vitamin a supplementation in very-low-birth-weight infants at risk for bronchopulmonary dysplasia. *Pediatric research* **1992**, *31*, 319A.

107. Plummer, E.L.; Bulach, D.M.; Murray, G.L.; Jacobs, S.E.; Tabrizi, S.N.; Garland, S.M. Gut microbiota of preterm infants supplemented with probiotics: sub-study of the ProPrems trial. *BMC microbiology* **2018**, *18*, 184, doi:10.1186/s12866-018-1326-1.

108. Puccio, G.; Cajozzo, C.; Meli, F.; Rochat, F.; Grathwohl, D.; Steenhout, P. Clinical evaluation of a new starter formula for infants containing live Bifidobacterium longum BL999 and prebiotics. *Nutrition (Burbank, Los Angeles County, Calif.)* **2007**, *23*, 1-8, doi:10.1016/j.nut.2006.09.007.

109. Qiao, L.X.; Zhu, W.Y.; Zhang, H.Y.; Wang, H. Effect of early administration of probiotics on gut microflora and feeding in pre-term infants: a randomized controlled trial. *The journal of maternal-fetal & neonatal medicine : the official journal of the European Association of Perinatal Medicine, the Federation of Asia and Oceania Perinatal Societies, the International Society of Perinatal Obstet* **2017**, *30*, 13-16, doi:10.3109/14767058.2016.1163674.

110. Radke, M.; Picaud, J.C.; Loui, A.; Cambonie, G.; Faas, D.; Lafeber, H.N.; de Groot, N.; Pecquet, S.S.; Steenhout, P.G.; Hascoet, J.M. Starter formula enriched in prebiotics and probiotics ensures normal growth of infants and promotes gut health: a randomized clinical trial. *Pediatric research* **2017**, *81*, 622-631, doi:10.1038/pr.2016.270.

111. Raguž, M.J.; Brzica, J.; Rozić, S.; Glamuzina, D.S.; Mustapić, A.; Bošnjak, M.N.; Božić, T. The impact of probiotics (Lactobacillus reuteri Protectis) on the treatment, course and outcome of premature infants in the Intensive Care Unit in Mostar. *Journal of Pediatric and Neonatal Individualized Medicine* **2016**, *5*, doi:10.7363/050228.

112. Rakshasbhuvankar, A.; Patole, S.; Simmer, K.; Pillow, J.J. Enteral vitamin A for reducing severity of bronchopulmonary dysplasia in extremely preterm infants: a randomised controlled trial. *BMC pediatrics* **2017**, *17*, 204, doi:10.1186/s12887-017-0958-x.

113. Rawat, M.C., P.; Turkovich, S.; Barclay, N.; Perry, K.; Schroeder, E.; Testa, L.; Lakshminrusimha,. Oral Dextrose Gel Reduces the Need for Intravenous Dextrose Therapy in Neonatal Hypoglycemia. *Biomed Hub* **Sep-Dec 2016**, *1*.

114. Repa, A.; Thanhaeuser, M.; Endress, D.; Weber, M.; Kreissl, A.; Binder, C.; Berger, A.; Haiden, N. Probiotics (Lactobacillus acidophilus and Bifidobacterium infantis) prevent NEC in VLBW infants fed breast milk but not formula [corrected]. *Pediatric research* **2015**, *77*, 381-388, doi:10.1038/pr.2014.192.

115. Robbins, S.T.; Fletcher, A.B. Early vs delayed vitamin A supplementation in very-low-birth-weight infants. *JPEN. Journal of parenteral and enteral nutrition* **1993**, *17*, 220-225, doi:10.1177/0148607193017003220.

116. Rodriguez-Herrera, A.; Mulder, K.; Bouritius, H.; Rubio, R.; Muñoz, A.; Agosti, M.; Lista, G.; Corvaglia, L.; Ludwig, T.; Abrahamse-Berkeveld, M., et al. Gastrointestinal Tolerance, Growth and Safety of a Partly Fermented Formula with Specific Prebiotics in Healthy Infants: A Double-Blind, Randomized, Controlled Trial. *Nutrients* **2019**, *11*, 1530-1530, doi:10.3390/nu11071530.

117. Rodirigez. Prevention of Necrotizing Enterocolitis of Premature Newborns Under Less Than 1500 g Using Probiotics.. <Https://clinicaltrials.gov/show/nct02245815> 2015. **2015**.

118. Rohan, A.J. Bifidobacterium Breve BBG-001 in Very Preterm Infants: A Randomised Controlled Phase 3 Trial. *MCN: The American Journal of Maternal Child Nursing* **2016**, *41*, 258-258, doi:10.1097/nmc.0000000000000253.

119. Rouge, C.; Piloquet, H.; Butel, M.J.; Berger, B.; Rochat, F.; Ferraris, L.; Des Robert, C.; Legrand, A.; de la Cochetiere, M.F.; N'Guyen, J.M., et al. Oral supplementation with probiotics in very-low-birth-weight preterm infants: a randomized, double-blind, placebo-controlled trial. *The American journal of clinical nutrition* **2009**, *89*, 1828-1835, doi:10.3945/ajcn.2008.26919.

120. Rubaltelli, F.; Biadaioli, R.; Dani, C. Probiotics feeding prevents necrotizing enterocolitis in preterm infants: a prospective double-blind study. *Pediatric research* **2000**, *47*, 346A.

121. Sadowska-Krawczenko, I.; Korbal, P.; Polak, A.; Wietlicka-Piszcz, M.; Szajewska, H. Lactobacilllus rhamnosus ATC A07FA for preventing necrotizing enterocolitis in very-low-birth-weight preterm infants: A randomized controlled trial (preliminary results). *Pediatria Polska* **2012**, *87*, 139-145, doi:10.1016/S0031-3939(12)70608-X.

122. Samuels, N.; van de Graaf, R.; Been, J.V.; de Jonge, R.C.; Hanff, L.M.; Wijnen, R.M.; Kornelisse, R.F.; Reiss, I.K.; Vermeulen, M.J. Necrotising enterocolitis and mortality in preterm infants after introduction of probiotics: a quasi-experimental study. *Scientific reports* **2016**, *6*, 31643, doi:10.1038/srep31643.

123. Shenai, J.P.; Kennedy, K.A.; Chytil, F.; Stahlman, M.T. Clinical trial of vitamin A supplementation in infants susceptible to bronchopulmonary dysplasia. *The Journal of pediatrics* **1987**, *111*, 269-277.

124. Smilowitz, J.T.; Lemay, D.G.; Kalanetra, K.M.; Chin, E.L.; Zivkovic, A.M.; Breck, M.A.; German, J.B.; Mills, D.A.; Slupsky, C.; Barile, D. Tolerability and safety of the intake of bovine milk oligosaccharides extracted from cheese whey in healthy human adults. *Journal of Nutritional Science* **2017**, *6*, doi:10.1017/jns.2017.2.

125. Storm, H.M.; Shepard, J.; Czerkies, L.M.; Kineman, B.; Cohen, S.S.; Reichert, H.; Carvalho, R. 2'-Fucosyllactose Is Well Tolerated in a 100% Whey, Partially Hydrolyzed Infant Formula With Bifidobacterium lactis: A Randomized Controlled Trial. *Global pediatric health* **2019**, *6*, 2333794x19833995, doi:10.1177/2333794x19833995.

126. Stratiki, Z.; Costalos, C.; Sevastiadou, S.; Kastanidou, O.; Skouroliakou, M.; Giakoumatou, A.; Petrohilou, V. The effect of a bifidobacter supplemented bovine milk on intestinal permeability of preterm infants. *Early human development* **2007**, *83*, 575-579, doi:10.1016/j.earlhumdev.2006.12.002.

127. Strus, M.; Helwich, E.; Lauterbach, R.; Rzepecka-Weglarz, B.; Nowicka, K.; Wilinska, M.; Szczapa, J.; Rudnicka, M.; Slawska, H.; Szczepanski, M., et al. Effects of oral probiotic supplementation on gut Lactobacillus and Bifidobacterium populations and the clinical status of low-birth-weight preterm neonates: a multicenter randomized, double-blind, placebo-controlled trial. *Infect Drug Resist* **2018**, *11*, 1557-1571, doi:10.2147/idr.S166348.

128. Ter, M.H., I.; Leung, L.; Jacobs, S. Implementation of dextrose gel in the management of neonatal hypoglycaemia. *J Paediatr Child Health* **Apr 2017**, *53*, 408-411.

129. Thanhaeuser, M.; Repa, A.; Weber, M.; Endress, D.; Kreissl, A.; Binder, C.; Berger, A.; Haiden, N. Probiotics (infloran®) for NEC prevention: Influence of enteral nutrition. *Archives of disease in childhood* **2014**, *99*, A176-A177, doi:10.1136/archdischild-2014-307384.478.

130. Totsu, S.; Yamasaki, C.; Terahara, M.; Uchiyama, A.; Kusuda, S. Bifidobacterium and enteral feeding in preterm infants: cluster-randomized trial. *Pediatrics international : official journal of the Japan Pediatric Society* **2014**, *56*, 714-719, doi:10.1111/ped.12330.

131. Tyson, J.E.; Wright, L.L.; Oh, W.; Kennedy, K.A.; Mele, L.; Ehrenkranz, R.A.; Stoll, B.J.; Lemons, J.A.; Stevenson, D.K.; Bauer, C.R., et al. Vitamin A supplementation for extremely-low-birth-weight infants. National Institute of Child Health and Human Development Neonatal Research Network. *The New England journal of medicine* **1999**, *340*, 1962-1968, doi:10.1056/nejm199906243402505.

132. Vitamin A supplementation in northern Ghana: effects on clinic attendances, hospital admissions, and child mortality. Ghana VAST Study Team. *Lancet (London, England)* **1993**, *342*, 7-12.

133. Venkatarao, T.; Ramakrishnan, R.; Nair, N.G.; Radhakrishnan, S.; Sundaramoorthy, L.; Koya, P.K.; Kumar, S.K. Effect of vitamin A supplementation to mother and infant on morbidity in infancy. *Indian pediatrics* **1996**, *33*, 279-286.

134. Vlieger, A.M.; Robroch, A.; van Buuren, S.; Kiers, J.; Rijkers, G.; Benninga, M.A.; te Biesebeke, R. Tolerance and safety of Lactobacillus paracasei ssp. paracasei in combination with Bifidobacterium animalis ssp. lactis in a prebiotic-containing infant formula: a randomised controlled trial. *The British journal of nutrition* **2009**, *102*, 869-875, doi:10.1017/s0007114509289069.

135. Wardle SP, H.A., Chen S, Shaw NJ. Randomised controlled trial of oral vitamin A supplementation in preterm infants to prevent chronic lung disease.=. *Archives of disease in childhood. Fetal and neonatal edition* **2001**, *84*, F9-F13.

136. West, K.P., Jr.; Pokhrel, R.P.; Katz, J.; LeClerq, S.C.; Khatry, S.K.; Shrestha, S.R.; Pradhan, E.K.; Tielsch, J.M.; Pandey, M.R.; Sommer, A. Efficacy of vitamin A in reducing preschool child mortality in Nepal. *Lancet (London, England)* **1991**, *338*, 67-71.

137. Yang S, Y.H., Gan B, et al. he clinical application value ofendangered preterm infants given earlier amounts of micro feedingsand adding probiotics. *J Pediat Pharmacy* **2011**, *17*.
